# Gamma Oscillations in the Basolateral Amygdala: Biophysical Mechanisms and Computational Consequences

**DOI:** 10.1523/ENEURO.0388-18.2018

**Published:** 2019-02-05

**Authors:** Feng Feng, Drew B. Headley, Alon Amir, Vasiliki Kanta, Ziao Chen, Denis Paré, Satish S. Nair

**Affiliations:** 1 Department of Electrical Engineering and Computer Science, and Department of Bioengineering, University of Missouri, Columbia, MO 65211; 2 Center for Molecular and Behavioral Neuroscience, Rutgers University – Newark, Newark, NJ 07102

**Keywords:** amygdala, gamma oscillations, computational model, biophysical model, extracellular potential

## Abstract

The basolateral nucleus of the amygdala (BL) is thought to support numerous emotional behaviors through specific microcircuits. These are often thought to be comprised of feedforward networks of principal cells (PNs) and interneurons. Neither well-understood nor often considered are recurrent and feedback connections, which likely engender oscillatory dynamics within BL. Indeed, oscillations in the gamma frequency range (40 − 100 Hz) are known to occur in the BL, and yet their origin and effect on local circuits remains unknown. To address this, we constructed a biophysically and anatomically detailed model of the rat BL and its local field potential (LFP) based on the physiological and anatomical literature, along with *in vivo* and *in vitro* data we collected on the activities of neurons within the rat BL. Remarkably, the model produced intermittent gamma oscillations (∼50 − 70 Hz) whose properties matched those recorded *in vivo*, including their entrainment of spiking. BL gamma-band oscillations were generated by the intrinsic circuitry, depending upon reciprocal interactions between PNs and fast-spiking interneurons (FSIs), while connections within these cell types affected the rhythm’s frequency. The model allowed us to conduct experimentally impossible tests to characterize the synaptic and spatial properties of gamma. The entrainment of individual neurons to gamma depended on the number of afferent connections they received, and gamma bursts were spatially restricted in the BL. Importantly, the gamma rhythm synchronized PNs and mediated competition between ensembles. Together, these results indicate that the recurrent connectivity of BL expands its computational and communication repertoire.

## Significance Statement

Using *in vitro* and *in vivo* data we develop the first large-scale biophysically and anatomically realistic model of the basolateral amygdala nucleus (BL), which reproduces the dynamics of the *in vivo* local field potential (LFP). Significantly, it predicts that BL intrinsically generates the transient gamma oscillations observed *in vivo*. The model permitted exploration of the poorly understood synaptic mechanisms underlying gamma genesis in BL, and the model's ability to compute LFPs at arbitrary numbers of recording sites provided insights into the characteristics of the spatial properties of gamma bursts. Furthermore, we show how gamma synchronizes principal cells (PNs) to overcome their low firing rates while simultaneously promoting competition, potentially impacting their afferent selectivity and efferent drive, and thus emotional behavior.

## Introduction

The basolateral complex of the amygdala (BLA) supports emotional learning and expression (Ehrlich et al., 2009; Duvarci and Pare, 2014). Central to this function is integrating information from numerous cortical and thalamic regions (Turner and Herkenham, 1991; McDonald, 1998). It is commonly thought that these signals converge on subpopulations of principal cells (PNs), which in turn either synapse on other PNs that promote a particular behavior, or contact interneurons that inhibit PNs supporting a countervailing behavior (LeDoux, 2000; Ehrlich et al., 2009; Duvarci and Pare, 2014). However, recurrent connections also exist within the BLA (Paré et al., 1995; Samson and Paré, 2006; Beyeler et al., 2016), with fast-spiking interneurons (FSIs) synapsing on each other and forming reciprocal connections with local PNs (Smith et al., 1998; Woodruff and Sah, 2007). Yet, the importance of these connections for BLA function remains largely unknown.

One possibility is that recurrent connections support the generation of oscillations. Consistent with this possibility, computational models (Traub et al., 1996; Wang and Buzsáki, 1996) as well as *in vitro* (Traub et al., 1996; Sohal et al., 2009) and *in vivo* experiments (Penttonen et al., 1998; Cardin et al., 2009) have revealed that a dense recurrent network of PNs and FSIs produces oscillations in the gamma frequency band. The dominant model for this, known as the pyramidal-interneuron network gamma (PING) model (Whittington et al., 2000), posits that the firing of PNs excites FSIs, which in turn deliver feedback inhibition, transiently silencing PNs. As the inhibition wanes, PNs regain the ability to fire and can restart the gamma cycle.

Crucially, during affective experiences, the BLA also exhibits gamma oscillations that are especially pronounced in its basolateral nucleus (BL; Bauer et al., 2007). For instance, gamma increases when rodents regulate their anxiety level during open field exploration (Stujenske et al., 2014) or are exposed to emotionally charged stimuli (Bauer et al., 2007). Importantly, the human amygdala also produces gamma oscillations during emotionally arousing stimuli (Oya et al., 2002). Despite the prevalence of gamma oscillations in the amygdala, their cellular basis and function remain unclear.

Numerous functions have been ascribed to gamma oscillations (Wang, 2010), but two stand out in particular. First, they synchronize spiking. PNs in networks exhibiting gamma oscillations tend to fire together more often than expected by chance (Wang and Buzsáki, 1996), robustly driving downstream neurons (Salinas and Sejnowski, 2000, 2002; Zandvakili and Kohn, 2015). Second, they may mediate competitive interactions between PN ensembles (Börgers et al., 2008). During each gamma cycle, the ensemble with the strongest afferent drive will tend to recruit the local FSI network, suppressing weakly driven ensembles (de Almeida et al., 2009).

Computational models of gamma oscillations have not been reported for the amygdala; such models for other brain regions have typically used generic single cell and network configurations, with surrogate local field potential (LFP) models (e.g., Börgers et al., 2008; Palmigiano et al., 2017). To examine whether the intrinsic BL circuitry can produce the poorly-understood transient gamma oscillations and their associated functions, we created a large-scale 27,000 cell multi-compartmental biophysical model of BL, with a detailed LFP model, that recapitulates numerous features of BL activity *in vivo*, such as the properties of gamma oscillations in LFPs and their entrainment of spiking. Using this model, we determined the circuit elements essential for generating gamma oscillations in BL, what aspects of microcircuit architecture affect the participation of neurons in gamma, the spatiotemporal properties of BL gamma bursts, and how these oscillations support information processing.

## Materials and Methods

### Experimental data

To construct and validate our model, we conducted new analyses on two sets of extracellular recordings that were described in prior publications (Headley et al., 2015; Amir et al., 2018), and on one unpublished dataset. Thus, we briefly describe the methods used in these prior studies so that readers can assess the nature and quality of these data.

#### *In vivo* chronic recordings

All animal procedures were approved by the Institutional Animal Care and Use Committee at Rutgers University, in accordance with the Guide for the Care and Use of Laboratory Animals (Department of Health and Human Services). Unit activity from prefrontal (PFC), perirhinal (PR), and entorhinal (ER) cortices (Headley et al., 2015) was used to generate surrogate spike trains that simulate extrinsic afferents onto the BL model (see below, BL afferents). These data were acquired in three male Long–Evans rats weighing between 350 and 500 g that were implanted with a headcap containing microdrives loaded with tetrodes (20-µm tungsten wire, impedance <100 kΩ). Two independent drives were used to target either prefrontal or PR/ER (PFC: AP +3.0, ML +0.5, DV 3.0; PR: AP −3.0 to −8.0, ML +6.0 to +7.2, DV 5.0; ER: AP −5.4 to −8.0, ML +7.0, DV 5.5; all coordinates in mm, DV was taken with respect to the pial surface). Following recovery from surgery (>7 d), microdrives were advanced until tetrodes reached their target locations, at which point recordings began. Extracellular signals were amplified with a 96-channel system (Plexon) and digitized (National Instruments) for offline analysis. Unit activity was sorted into single units by high pass filtering wideband LFP with a moving median filter, detection of spikes with amplitude >2 SD, automatic clustering of waveforms in principal component space (KlustaKwik), and manual refinement of cluster assignment (Klusters). Validation of single unit quality and isolation can be found in our prior paper (Headley et al., 2015). Only regular spiking units (putative projection neurons), classified using k-means clustering of the negative peak to positive peak time interval of their waveform and firing rate, were used. In particular, we focused on a single epoch from each subject (subject 1: 165 s; subject 2: 140 s; subject 3: 160 s) with simultaneous recordings from multiple single units (subject 1: 36; subject 2: 14; subject 3: 20).

Unit and LFP activities from the BL recorded with silicon probes were used to calibrate and validate our model (*n* = 5). As detailed in Amir et al. (2018), male Sprague Dawley rats (>350 g) were implanted with either a 32 or 64 channel silicon probe (Neuronexus) mounted on a microdrive and targeting the BL nucleus of the amygdala (AP −2.2 to −3.6, ML +5 − 5.3, and DV 8.8). Recording hardware and single unit sorting was similar to that described above.

LFP spectra were also recorded from the BL of male Long–Evans rats (>350 g, *n* = 7). A microwire (20-µm tungsten wire, impedance <50 kΩ) attached to a fiber optic stub was stereotaxically implanted in the BLA (AP −2.5, ML +5.0, DV −7.5, from brain surface) and fixed to the skull with dental cement (Metabond and Teets Cold Cure). These subjects were also used for optogenetic experiments (AAV5-Syn-Chronos-GFP injected into BL), but these data were excluded from the present study. All recordings were performed at least 24 h following any optogenetic manipulations. Extracellular recordings from the BL were obtained with a 32-channel digitizing headstage (Intan Technologies).

All the electrophysiological data used in the present study was referenced to a screw fixed to the bone overlaying the cerebellum and was obtained while rats were allowed to behave spontaneously in a neutral plastic enclosure. We only considered data acquired in the quiet waking state (QW), which was identified by the absence of gross body movement and LFPs with relatively low power at frequencies <4 Hz.

#### *In vitro* BL slice recordings

An overdose of isoflurane was administered to deeply anesthetize male Long–Evans rats (*n* = 11, 53 cells). Once all reflexes had ceased, they were immediately perfused through the heart with a ice cold modified artificial CSF (aCSF) solution containing: 103 mM N-methyl-D-gluconamine, 2.5 mM KCl, 1.2 mM NaH_2_PO_4_, 30 mM NaHCO_3_, 10 mM MgSO_4_ × 7H_2_O, 25 mM glucose, 20 mM HEPES, 101 mM HCl, 2 mM Thiourea, 3 mM Na-Pyruvate, 12 mM N-acetyl-L-cysteine, and 0.5 mM CaCl. Following 30 s of perfusion, the brain was rapidly extracted and placed in the cutting solution, which was the same used for perfusion. Slices were cut in the coronal plane on a vibratome with a thickness of 300 − 400 µm. Following slicing sections were transferred to a chamber containing the perfusion solution at 32° C for 5 min. Slices were then placed in a holding chamber with room temperature (22°C) normal aCSF: 124 mM NaCl, 2.5 mM KCl, 1.25 mM NaH_2_PO_4_, 26 mM NaHCO_3_, 1 mM MgCl_2_, 2 mM CaCl_2_, and 10 mM glucose (pH 7.2 − 7.3, 305 mOsm). Slices were then transferred one at a time to a recording chamber perfused with oxygenated aCSF at 32°C.

Under visual guidance by infrared video microscopy, we obtained whole-cell patch-clamp recordings from BL neurons. We pulled patch pipettes of borosilicate glass with tip resistances of 5 − 8 MΩ and filled them with a solution of 130 mM K-gluconate, 10 mM N-2-hydroxyethylpiperazine-N’-2’-ethanesulfonic acid, 10 mM KCl, 2 mM MgCl_2_, 2 mM ATP-Mg, and 0.2 mM GTP-tris(hydroxy-methyl)aminomethane (pH 7.2, 280 mOsm). We did not compensate for the liquid junction potential, which is 10 mV with this solution. Current clamp recordings were obtained with a MultiClamp 700B amplifier and digitized at 20 kHz using an Axon Digidata 1550 interface.

Once whole-cell access was achieved, we characterized the neuron’s electroresponsive properties. Current pulses 500 ms in length were delivered from −200 to 360 pA in steps of 40 pA. Neurons with action potential (AP) half-widths <0.35 ms were classified as FSIs, while all others were regular spiking.

### Model implementation

**Figure 1.**
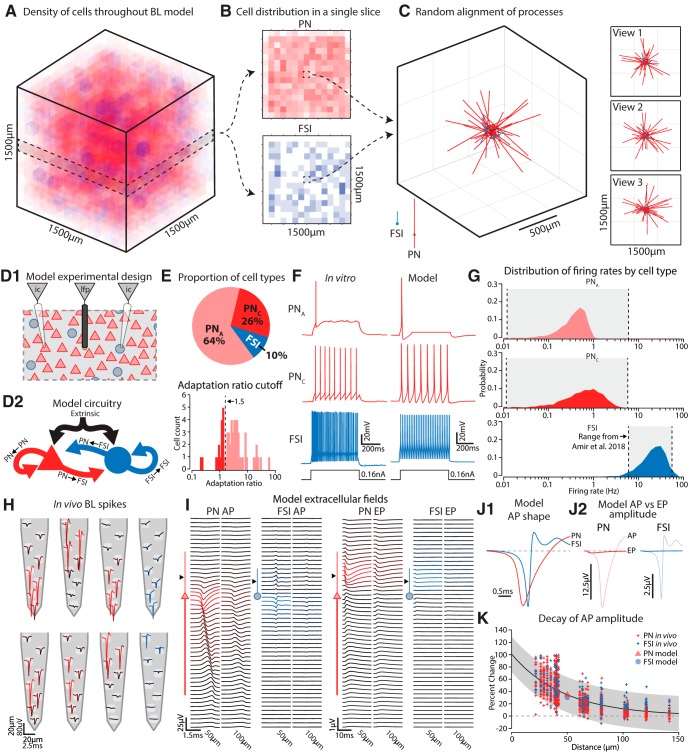
Construction of a biophysically accurate BL model. ***A***, Our BL model was a cube structure comprised of 27,000 PNs and FSIs randomly distributed throughout. Local cell densities in 100 × 100 × 100 µm cubes (red = PN, blue = FSI) are illustrated for a typical case. For this example, PNs, the densities spanned 0–19,000/mm^3^ (median = 7000/mm^3^). FSI densities ranged from 0 to 5000/mm^3^ (median = 1000/mm^3^). ***B***, A slice through the model shows the inhomogeneity of cell densities for PNs and FSIs. ***C***, The dendritic processes of the neurons contained in a particular voxel are illustrated. These were randomly oriented and spanned several hundred microns as also shown in the three orthogonal views. ***D***, (1) PNs and FSIs were distributed in space so that they spanned a volume equal to the area of the BL in the rat. Virtual current clamp electrodes (ic) could be placed into any cell, and virtual extracellular electrodes (lfp) could be placed anywhere in the model volume. PNs are indicated by red triangles and FSIs are blue circles. (2) PNs and FSIs were connected among themselves and with each other. Extrinsic glutamatergic afferents fed onto both cell types. Neither FSIs nor PNs formed autapses. ***E***, The relative proportions of the three cell types were determined by patching neurons in BL slices prepared from adult Long–Evans rats, the same age and strain used for our LFP recordings. The cutoff for determining which PNs exhibited adaption was set to 1.5, which was between the two peaks in the distribution of adaptation ratios. ***F***, Example recordings from neurons in the slice receiving current injection were comparable to those of our model neurons. ***G***, The firing rate distributions for neurons in our model overlapped with the mean rates reported from the BL *in vivo* in a previous study (Amir et al., 2018). ***H***, Example spike waveforms recorded with a silicon probe *in vivo*. Red traces are from putative PNs, while blue are FSIs. The intensity of the color is scaled to the peak of the spike wave form. ***I***, Left, For both a model PN and FSI we delivered a suprathreshold EPSC to the a-dend and recorded the extracellular AP at different distances along the long axis of the neuron, and either at 50 or 100 µm lateral. Near the cell body the field was negative, and it decayed rapidly with distance. Positive dendritic return currents were evident as well. The FSI extracellular spike wave form was both smaller and faster than the PN’s. Right, Subthreshold stimulation resulted in a much weaker extracellular wave form (note the scale bar) reflecting the EPSC (EP) that was negative going near the stimulated dendritic branch. ***J***, (1) Directly overlaying the extracellular APs from both cell types illustrates that those arising from PNs were much slower than those from FSIs. Amplitudes were rescaled so both spike waveforms occupy the same vertical extent of the graph. (2) AP amplitudes were much stronger than EP amplitudes for both cell types. ***K***, For extracellular APs recorded with silicon probes *in vivo*, we measured how their amplitudes decayed with distance. The drop in amplitude was fit by an exponential curve (black line). The gray region is the 95% confidence bounds. Measuring the decay in our model extracellular APs along the lateral axis, we found that it fit within the *in vivo* distribution.

**Figure 2.**
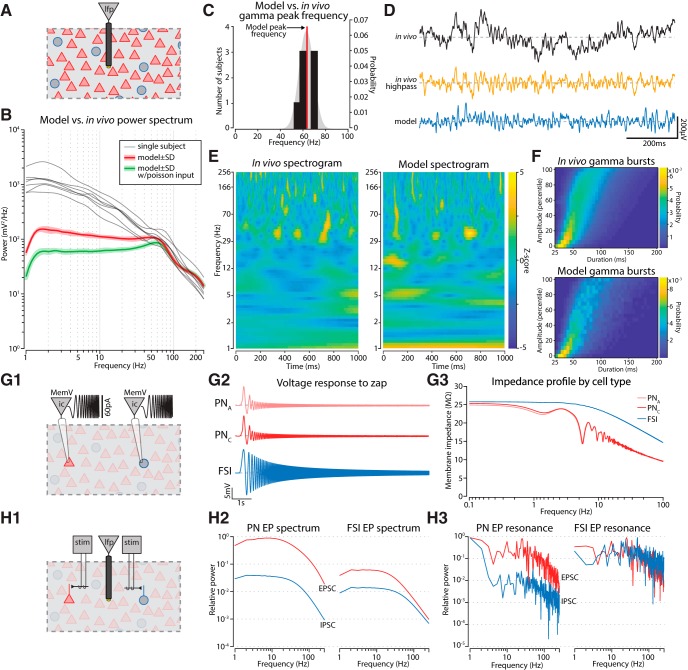
The model LFP exhibits gamma oscillations that are similar to those seen *in vivo*. ***A***, The LFP was measured at the center of the model. ***B***, The power spectrum of the model LFP (red) is compared with the spectra from the BL of seven rats during QW (gray). Driving the model with a homogenous Poisson input (green) still induces gamma oscillations. Shaded regions denote SD. ***C***, The frequency where the gamma bump peaked was measured for each subject (black bars), the probability distribution was fit with a normal curve (gray). The model’s peak frequency was 64 Hz (red line), which fell within the *in vivo* distribution. ***D***, Gamma oscillations occurred as intermittent bursts *in vivo* and in the model. There was a stronger low-frequency component *in vivo* (black), but filtering that out (yellow) revealed a comparable signal amplitude to our model (blue). ***E***, The model had a similar wavelet spectrogram to that observed *in vivo*. ***F***, Gamma bursts were detected in the wavelet power spectrograms and categorized based on their amplitude percentile and duration. The relationship between these features was characterized by a probability distribution. Bursts arising from the model or *in vivo* exhibited a similar distribution of durations when stratified by amplitude, with higher amplitude bursts tending to last longer. ***G***, (1) Each simulated cell type was placed in current clamp and driven with a frequency modulated sinusoid. (2) Neurons responded to this input with an oscillating membrane potential that decayed with increasing frequency. (3) The impedance spectrum of the neuronal responses did not exhibit any peaks in the gamma band. ***H***, (1) An extracellular electrode was placed near either a PN or FSI and synaptic inputs to those neurons were driven (stim). (2) The extracellular field responding arising from a single EPSC or IPSC was measured for each cell, and normalized to the strength of the PN EPSC. There was no bump in the gamma band. (3) Driving the synaptic inputs with Poisson trains at rates indicated by the *x*-axis did not show any obvious boost in power in the gamma range either.

**Figure 3.**
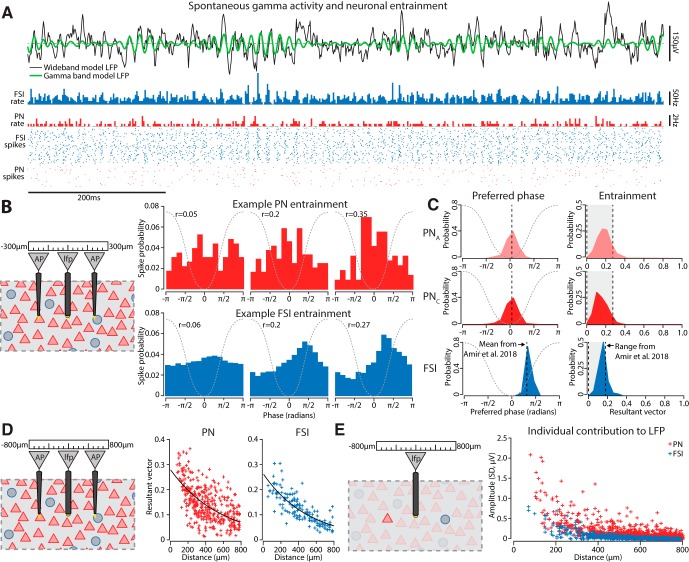
Spiking in the model was entrained to the gamma rhythm similarly to cells *in vivo*. ***A***, Example trace of the calculated extracellular field from the center of the model and a raster plot of corresponding spiking from neurons within 300 µm (1026 PNs, 111 FSIs). Gamma bursts were evident, particularly following bandpass filtering around the peak gamma frequency. The group firing rates of FSIs and PNs exhibited modulations by the gamma rhythm, with the effect being more evident for FSIs. Spikes from individual neurons (each row is a different cell) did not spike on every gamma cycle. ***B***, Left, Spiking from units (AP) within 300 µm of where the extracellular field was measured (LFP) were associated with the phase of the ongoing gamma oscillations. Right, Distribution of gamma phases when spikes occurred for three example PNs and FSIs, sorted by the strength of their phase locking. Dashed gray schematizes the corresponding gamma cycle. ***C***, Left, The distribution of mean phases across each cell type. Both types of PNs preferentially spiked near the trough of the gamma oscillation, while FSIs spiked shortly thereafter during the ascending phase. Right, The distribution of phase-locking across cell types matched what we found for BL neurons *in vivo*. ***D***, Left, Spiking was sampled from neurons spanning 800 µm away from the point where we measured the extracellular field, and neurons were sorted by their distance from that spot. Right, The farther a neuron was from the site were gamma was measured, the less phase-locked it was. Black lines are exponential fits. ***E***, Left, The extracellular field arising from individual neurons was singled out to examine their distance-dependent contribution. Right, Neurons farther from the recording site contributed less to the extracellular field, and this fell off faster than the decay in the resultant vector in panel ***D***.

**Figure 4.**
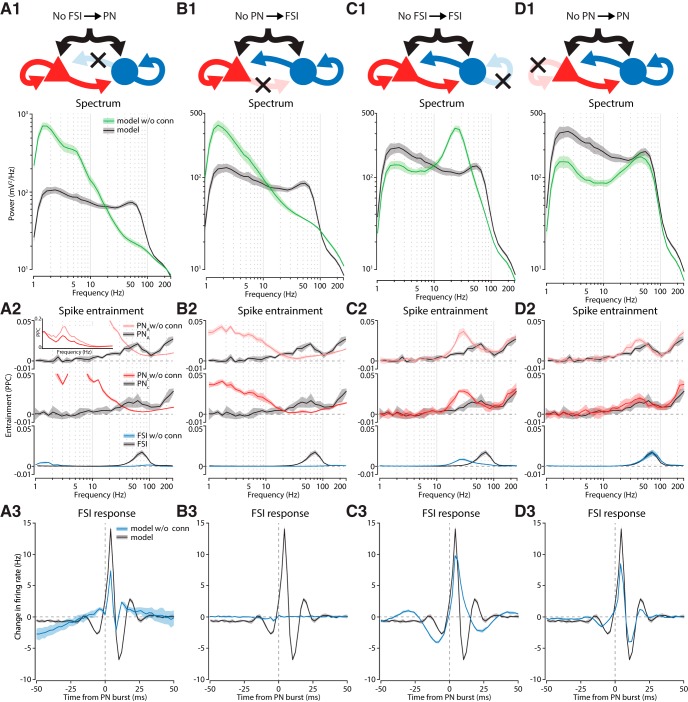
Connections between PNs and FSIs were critical for genesis of the gamma rhythm. ***A***, Removal of connections from FSIs to PNs. (1) Comparison of power spectrum between the original and perturbed model. Gray traces are derived from the unmodified model and are the same across all graphs in each row. The green traces are the power spectrum with the particular connection removed. (2) Entrainment to LFP oscillations for different cell types. (3) Average modulation of FSI firing rate triggered on PN population bursts. ***B***, Removal of connections from PNs to FSIs. ***C***, Removal of connections between FSIs. ***D***, Removal of connections between PNs. For all graphs the solid line is the mean while the shaded region denotes ±1 SD.

**Figure 5.**
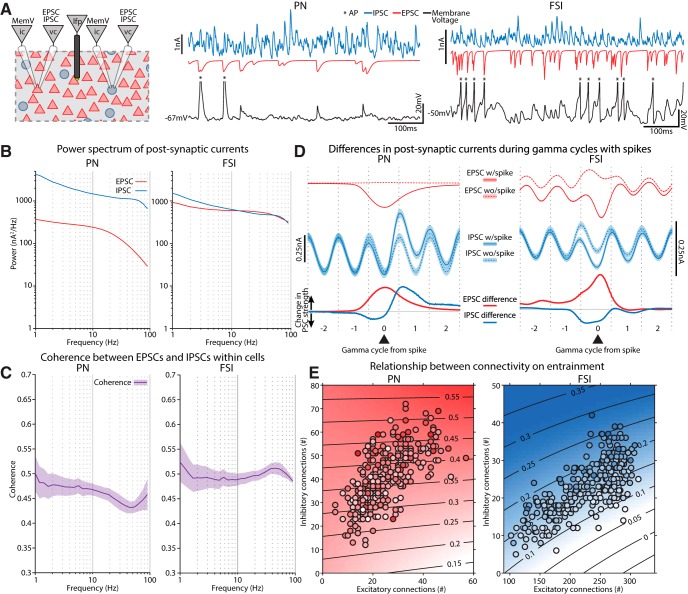
EPSCs and IPSCs entrained neurons in the gamma band. ***A***, Left, For PNs and FSIs we measured the membrane voltage (ic) and their transmembrane currents while voltage clamped (vc) at the reversal potentials for either EPSCs (0 mV) or IPSCs (−75 mV). Middle, Traces from an example PN. In addition to the transmembrane currents and voltages were recorded, we also bandpass filtered the EPSC and IPSC traces around the gamma peak. Right, Examples traces from an FSI. ***B***, The mean power spectrums of the EPSC and IPSC traces for each cell type. PNs showed greater power for IPSCs across all frequencies compared with EPSCs. FSIs had similar power levels across the spectrum for EPSCs and IPSCs. The spectrum of IPSCs from both cell types contained a bump in the gamma band. ***C***, The coherence of the EPSCs and IPSCs for each cell type. PNs had a dip in coherence around the peak gamma frequency, while FSIs showed a prominence. Shaded regions denote SD. ***D***, Synaptic currents were aligned to the phase of gamma cycles and centered on ones that either had, or did not have, spiking (thin blue and red lines, solid lines from cycles with spikes, dashed for cycles without spikes). The difference in the PSCs between the spike and no spike conditions is shown below (thick red and blue lines). IPSCs were modulated by gamma phase for both cell types, while only FSIs exhibited modulation of their EPSCs to gamma. ***E***, For each cell we plotted the number of excitatory (*x*-axis) or inhibitory (*y*-axis) connections they received and their corresponding entrainment. Each scatter point is a different cell. The shaded background is the result of a linear fit that estimates the resultant vector strength based on the number of inhibitory connections, excitatory connections, and their product (interaction term). Contours in black indicate the actual resultant vector values associated with each color.

**Figure 6.**
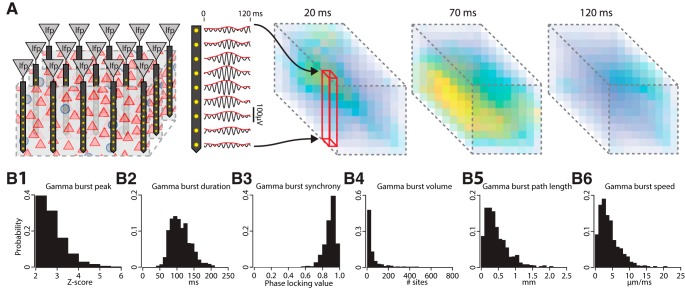
Spatiotemporal properties of gamma bursts. ***A***, Left, A 9 × 9 × 9 array of electrodes was inserted into the model and the envelope of the gamma band was extracted. Right, A cube of amplitude values was generated and short lasting, spatially localized, increases in gamma power were readily evident. ***B***, We examined the properties of these bursts, in particular their (1) amplitude, (2) duration, (3) phase synchrony at their peak, (4) number of sites they encompassed, (5) length of the path they took through the BL from initiation to termination, and (6) speed that they traversed that path.

**Figure 7.**
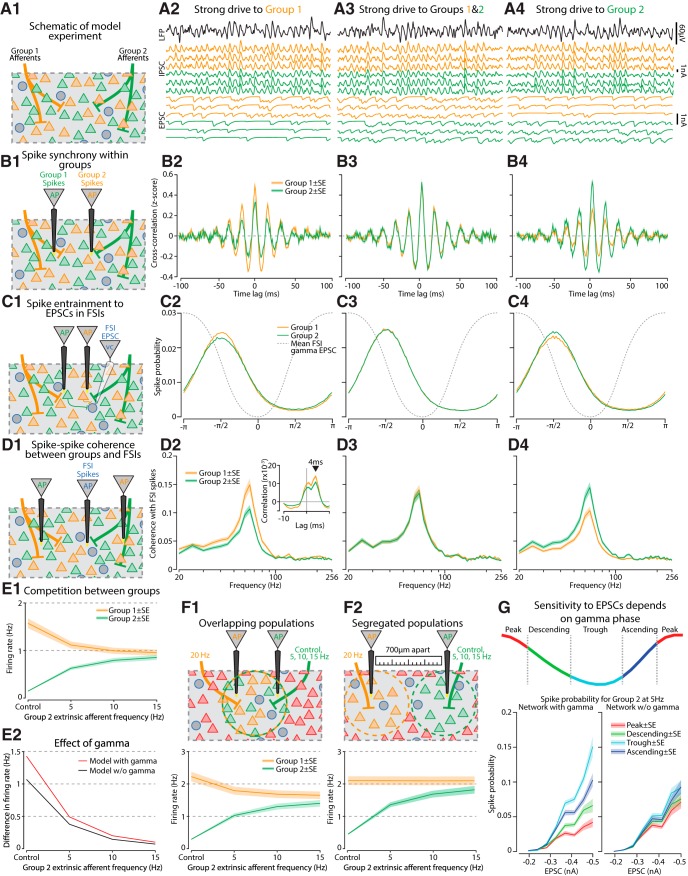
Gamma promoted synchrony between PNs responding to extrinsic afferents and the competition between weakly and strongly driven populations. ***A***, Two separate ensembles of PNs were targeted by extrinsic afferents (group 1, orange vs group 2, green), that received different subsets of afferents. Groups could be driven independently, with (2) group 1 receiving 20-Hz stimulation while group 2 had 5 Hz; (3) group 1, 20 Hz and group 2, 20 Hz; or (4) group 1, 5 Hz and group 2, 20 Hz. ***B***, (1) Spikes were recorded from both groups of PNs and (2–4) we calculated their cross-correlation functions. ***C***, (1) Spikes from PNs and the mean EPSC across all FSIs were recorded. (2–4) Probability histograms of the EPSC gamma phases associated with spiking from either group 1 or group 2. The strongly driven group exhibits greater entrainment. ***D***, (1) The coherence between PN spiking and FSI spiking for each group. (2–4) FSIs were more coherent in the gamma-band with the population that received the strongest afferent drive. Inset, PN preceded FSI spiking by 4 ms on average. ***E***, (1) Group 1 receives 20-Hz extrinsic drive while group 2 receives a varying amount. Increasing the firing rate of extrinsic afferents onto group 2 diminished the firing rate of group 1. (2) The difference in firing rate between these groups was weakened when connections from PNs to FSIs were removed. ***F***, Analysis of competition between groups in the full model when they either (1) fully overlap or (2) are entirely segregated in space. ***G***, top, Gamma cycles were divided into four phases and for each phase the spike probability was measured as a function of the EPSC strength. Bottom left, For the weakly driven group there was increased sensitivity for EPSCs during the trough and ascending phases of gamma, but a dampening of responsiveness during the descending and peak phases. Bottom right, When connections from PNs to FSIs were removed, the discrepant sensitivities for EPSCs across the four phases were diminished. Graphs are either mean or mean ± SE when a shaded region is present.

The single cell and network models were developed using the parallel NEURON 7.4 simulator (Carnevale and Hines, 2005), and simulations were run with a fixed time step of 50 µs. Network models were run using the NeuroScience Gateway (NSG; www.nsgportal.org) that provides free and easy access to high-performance computers (Sivagnanam et al., 2013). Model results were obtained by averaging five network model runs with different random seeds for data shown in Figures 1*G*, 2*B*, 3*C*, 4, 5*E* and 6*B1-B6* and for selecting groups of neurons were used in Figure 7*B–D*.

#### Mathematical equations for voltage-dependent ionic currents

The equation for each compartment (soma or dendrite) followed the Hodgkin-Huxley formulation (Byrne and Roberts, 2004; Kim et al., 2013) in Equation 1,

(1) $$C_{m}dV_{s}/dt=-g_{L}\left(V_{s}-E_{L}\right)-g_{c}\left(V_{s}-V_{d}\right)-\sum I_{cur,s}^{int}-\sum I_{cur,s}^{syn}+I_{inj}$$

where $V_{s}/V_{d}$ are the somatic/dendritic membrane potential (mV), $I_{cur,s}^{int}$ and $I_{cur,s}^{syn}$ are the intrinsic and synaptic currents in the soma, $I_{inj}$ is the electrode current applied to the soma, $C_{m}$ is the membrane capacitance, $g_{L}$ is the is the conductance of leak channel, and $g_{c}$ is the coupling conductance between the soma and the dendrite (similar term added for other dendrites connected to the soma). The intrinsic current $I_{cur,s}^{int}$, was modeled as $I_{cur,s}^{int}=g_{cur}m^{p}h^{q}(V_{s}-E_{cur})$, where $g_{cur}$ is its maximal conductance, *m* its activation variable (with exponent *p*), *h* its inactivation variable (with exponent *q*), and $E_{cur}$ its reversal potential (a similar equation is used for the synaptic current $I_{cur,s}^{syn}$ but without *m* and *h*). The kinetic equation for each of the gating variables *x* (*m* or *h*) takes the form

(2) $$\frac{dx}{dt}=\frac{x_{\infty }\left(V,{\left[{Ca}^{2+}\right]}_{i}\right)-x}{\tau_{x}(V,{\left[{Ca}^{2+}\right]}_{i})}$$

where $x_{\infty }$ is the steady state gating voltage- and/or Ca^2+^-dependent gating variable and $\tau_{x}$is the voltage**-** and/or Ca^2+^**-**dependent time constant. The equation for the dendrite follows the same format with ‘*s*’ and ‘*d*’ switching positions in Equation 1.

#### PN models

To reproduce the range of spike frequency adaptation exhibited by BL PNs (Rainnie et al., 1993), we modeled two types of PNs, one with adaptation (Type-A) and one with continuous spiking (Type-C), differing solely in the magnitude of their Ca^2+^-dependent K^+^ current, either 50 or 0.2 mS/cm^2^, respectively (Kim et al., 2013). We conducted our own study of the relative proportion of these types because our *in vivo* recordings, which our model was compared with, were from adult rats (>350 g) whereas previous slice work was conducted in younger subjects, and the ionic mechanisms underlying accommodation, such as spike after-hyperpolarizations (AHPs), change with age (Disterhoft and Oh, 2007). Positive current injection into PNs (*n* = 56) evoked either a train of APs with increasing interspike intervals (i.e., accommodation) or at a fixed rate (i.e., continuous). This property could be quantified by the adaptation ratio:

(3) $$adaptation\;ratio=\frac{duration\;of\;last\;ISI}{duration\;of\;first\;ISI}$$

with larger values indicating greater adaptation. We set a cutoff of 1.5 to classify PNs adapting. Our previous PN models (Li et al., 2009; Kim et al., 2013; Feng et al., 2016) were revised to incorporate the low-threshold and high-threshold oscillations (LTOs and HTOs, respectively) reported in BLA PNs (Pape et al., 1998) and modeled recently by our group (Alturki et al., 2016). The model PNs had three compartments representing a soma (diameter, 24.75 µm; length, 25 µm), where GABAergic synapses were placed, an apical dendrite (a-dend; diameter, 3 µm and length, 270 µm) where glutamatergic synapses were located, and another dendrite (p-dend; diameter, 5 µm and length, 555 µm) to match passive properties. Values of specific membrane resistance, membrane capacity and cytoplasmic (axial) resistivity were, respectively, R_m_ = 55 kΩ-cm^2^, C_m_ = 2.4 µF/cm^2^, and R_a_ = 150 Ω-cm. Leakage reversal potential (*E*_L_) was set to −75 mV. The resulting V_rest_ was −70.3 mV, input resistance (*R*_IN_) was ∼140 MΩ, and time constant (τ_m_) was ∼30 ms, all of which were within the ranges reported in previous physiologic studies (Washburn and Moises, 1992). All compartments had the following currents: leak (*I*_L_), voltage-gated persistent muscarinic (*I*_M_), high-voltage activated Ca^2+^ (*I*_Ca_), spike-generating sodium (*I*_Na_), potassium delayed rectifier (*I*_DR_), A-type potassium (*I*_A_; Li et al., 2009; Power et al., 2011) and hyperpolarization-activated nonspecific cation (*I*_h_) current. In addition, the soma had a slow apamin-insensitive, voltage-independent AHP current (*I*_sAHP_; Power et al., 2011; Alturki et al., 2016). See Tables 1, 2 for current equations and densities.

**Table 1.** Gating parameters of ion channels in BL PN neurons

Current type	Gating variable	α	β	$x_{\infty }$	τ_x_ (ms)
I_Na_	*p* = 3	$\frac{-0.4(V+30)}{exp[-(V+30)/7.2]-1}$	$\frac{0.124(V+30)}{exp[(V+30)/7.2]-1}$	$\frac{\alpha }{\alpha +\beta }$	$\frac{0.6156}{\alpha +\beta }$
	*q* = 1	$\frac{-0.03(V+45)}{exp[-(V+45)/1.5]-1}$	$\frac{0.01(V+45)}{exp[(V+45)/1.5]-1}$	$\frac{1}{\exp\left[(V+50)/4\right]+1}$	$\frac{0.6156}{\alpha +\beta }$
*I_Kdr_*	*p* = 1	exp[-0.1144(V+15)]	exp[-0.0801(V+15)]	$\frac{1}{\exp\left[(-V-15)/11\right]+1}$	$\frac{50*\beta }{1+\alpha }$
*I_H_*	*q* = 1	exp[0.0832(V+75)]	$exp[0.0333\left(V+75\right)]$	$\frac{1}{\exp\left[(V+81)/8\right]+1}$	$\frac{\beta }{0.0081(1+\alpha )}$
*I_KM_*	*p* = 2	$\frac{0.016}{exp[-(V+52.7)/23]}$	$\frac{0.016}{exp[(V+52.7)/18.8]}$	$\frac{1}{\exp\left[(-V-52.7)/10.3\right]+1}$	$\frac{1}{\alpha +\beta }$
*I_Ca_*	*p* = 2	―	―	$\frac{1}{\exp\left[(-V-30)/11\right]+1}$	$\frac{2.5}{\exp\left[\frac{-\left(V+37.1\right)}{32.3}\right]+\exp\left[\frac{\left(V+37.1\right)}{32.3}\right]}$
	*q* = 1	―	―	$\frac{1}{\exp\left[(V+12.6)/18.9\right]+1}$	420
*I_Nap_*	*p* = 1	―	―	$\frac{1}{\exp\left[(-V-48)/5\right]+1}$	$2.5+14*\exp\left[-\left|V+40\right|/10\right]$
*I_sAHP_*	*p* = 1	$\frac{0.0048}{exp[-5\log_{10}\left({\left[Ca\right]}_{i2})-17.5\right]}$	$\frac{0.012}{exp[2\log_{10}\left({\left[Ca\right]}_{i2}\right)+20]}$	$\frac{\alpha }{\alpha +\beta }$	48

**Table 2.** Maximal conductance densities in model BL PN neurons

Conductance (mS/cm^2^)		*I*_Na_	*I*_DR_	*I*_M_	*I*_H_	*I*_Ca_	*I_Nap_*	*I*_A_	*I*_sAHP_	*I_leak_*	*τ*_Ca_ (ms)
3-comp PN model	Soma	45	2	2.24	0.015	0.55	0.559	2	50/0.2 Types: A/C	0.025	1000
	Prox. P_dend	45	2	1.792	0.015	0.55	0.447	-	-	0.0471	-
	P_dend	45	2	-	0.015	0.55	-	-	-	0.0471	-
	Prox. A_dend	45	2	2.24	0.015	0.55	0.559	2	-	0.0471	-
	A_dend	45	2	-	0.015	0.55	-	2	-	0.0471	-

#### Interneuron models

BL also contains local GABAergic interneurons that exhibit various firing patterns, even among neurochemically-homogeneous subgroups (Pape and Pare, 2010; Spampanato et al., 2011). However, the most prevalent are the fast-spiking parvalbumin-positive type, which has been implicated in the genesis of cortical and BL gamma (Börgers et al., 2005; Oren et al., 2006; Atallah and Scanziani, 2009; Amir et al., 2018). Accordingly, we modeled only the fast-spiking type of interneurons (FSI). The FSI model was the same as in Kim et al., 2013, with two compartments, a soma (diameter, 15 µm and length, 15 µm) and a dendrite (diameter, 10 µm and length, 150 µm). Each compartment contained a fast Na^+^ (*I*_Na_) and a delayed rectifier K^+^ (*I*_DR_) current. The FSI model reproduced the short spike duration (with the spike duration at half amplitude <1 ms) that characterizes FSIs. The passive membrane properties of FSI cells were as follows: R_m_ = 20 kΩ-cm^2^, C_m_ = 1.0 µF/cm^2^, R_a_ = 150 Ω-cm. The FSI model also reproduced the non-adapting repetitive firing behavior of fast-spiking cells, as observed experimentally (Rainnie et al., 1993; Woodruff and Sah, 2007).

#### Network size and cell type proportions

Estimates of the number of neurons in rat BL vary widely (Chareyron et al., 2011), so we settled on the mean across studies, which was ∼72,000. We developed a scaled down (1:2.7) model of this region with 27,000 neurons randomly distributed in a cuboid geometry (1.4 × 1.4 × 1.4 mm), ensuring an intersoma distance >25 µm. The model included 64% PN_A_ (*n* = 17,280), 26% PN_C_ (*n* = 7020), and 10% FSIs. These proportions were based on *in vitro* results collected for this study and agreed with estimates found in the literature (McDonald and Mascagni, 2001; Muller et al., 2006). The dendrites of all neurons in the network had random orientations (McDonald, 1984).

#### Mathematical equations for synaptic currents

All excitatory transmission was mediated by AMPA/NMDA receptors, and inhibitory transmission by GABA_A_ receptors. The corresponding synaptic currents were modeled by dual exponential functions (Destexhe et al., 1994; Durstewitz et al., 2000), as shown in Equations 4–6,

$$I_{AMPA}=w*G_{AMPA}*(V-E_{AMPA})$$

$$G_{AMPA}=g_{AMPA,max}*{STP}_{AMPA}*r_{AMPA}$$

(4) $$r_{AMPA}\text{′}={\alpha Tmax}_{AMPA}*{ON}_{AMPA}*\left(1-r_{AMPA}\right)-\beta_{AMPA}*r_{AMPA}$$

$$I_{NMDA}=w*G_{NMDA}*(V-E_{NMDA})$$

$$G_{NMDA}=g_{NMDA,max}*{STP}_{NMDA}*s\left(V\right)*r_{NMDA}$$

(5) $$r_{NMDA}\text{′}={\alpha Tmax}_{NMDA}*{ON}_{NMDA}*\left(1-r_{NMDA}\right)-\beta_{NMDA}*r_{NMDA}$$

$$I_{GABAa}=w*G_{GABAa}*(V-E_{GABAa})$$

$$G_{GABAa}=g_{GABAa,max}*{STP}_{GABAa}*r_{GABAa}$$

(6) $$r_{GABAa}\text{′}={\alpha Tmax}_{GABAa}*{ON}_{GABAa}*\left(1-r_{GABAa}\right)-\beta_{GABAa}*r_{GABAa}$$

where *V* is the membrane potential (mV) of the compartment (dendrite or soma) where the synapse is located and w is the synaptic weight for the synapse. The synaptic reversal potentials were *E*_AMPA_ = *E*_NMDA_ = 0 mV and *E*_GABAa_ = −75 mV (Durstewitz et al., 2000; Martina et al., 2001). The voltage-dependent variable *s*(*V*) which implements the Mg^2+^ block was defined as: *s*(*V*) = [1 + 0.33 exp(−0.06 V)]^−1^ (Zador et al., 1990). The terms *ON_NMDA_* and *ON_AMPA_* are set to 1 if the corresponding receptor is open, else to 0. The synaptic current rise and decay time constants are determined by *αTmax* and *β* (Destexhe et al., 1994). Synaptic parameter values are listed in Table 3.

**Table 3.** Parameters related to synaptic connections

Parameters Connection type	AMPA				NMDA				GABA			
	Reversal potential (mV)	Rise/decay time constant (ms)	Conductance (nS)	Strength (mean/var.)	Reversal potential (mV)	Rise/decay time constant (ms)	Conductance (nS)	Strength (mean/var.)	Reversal potential (mV)	Rise/decay time constant (ms)	Conductance (nS)	Strength (mean/var.)
PN to PN	0	0.3/6.9 (Mahanty and Sah, 1998; Guzman et al., 2016)	1	5/3	0	3.7/125 (Weisskopf et al., 1999)	0.5	2/1	--	--	--	--
PN to FSI	0	0.1/2.4 (Mahanty and Sah, 1998; Guzman et al., 2016)	1	7/2	0	3.7/125 (Weisskopf et al., 1999)	0.5	7/2	--	--	--	--
FSI to PN	--	--	--	--	--	--	--	--	−75	0.5/6.80 (Galarreta and Hestrin, 1997)	0.6	12/2
FSI to FSI	--	--	--	--	--	--	--	--	−75	0.5/6.80 (Galarreta and Hestrin, 1997)	0.2	20/10
FSI to FSI (gap junction)	--				--				Gap junction coupling coefficient			
									∼0.05 (Woodruff and Sah, 2007)			

#### Short-term presynaptic plasticity

All model AMPA and GABA synapses also exhibited short term pre-synaptic plasticity (Kim et al., 2013). Short-term depression was modeled at FSI→PN and PN→FIS connections based on experimental findings of Woodruff and Sah (2007) in BL, while between PNs, it was modeled based on results from neocortex (Silberberg et al., 2004) due to the lack of such experimental data in BL. Short term plasticity was implemented as follows (Hummos et al., 2014): for facilitation, the factor F was calculated using the equation: $\tau_{F}*\frac{dF}{dt}=1-F$ and was constrained to be ≥1. After each stimulus, F was multiplied by a constant, f (≥1) representing the amount of facilitation per pre-synaptic AP, and updated as F→F*f. Between stimuli, *F* recovered exponentially back toward 1. A similar scheme was used to calculate the factor D for depression: $\tau_{D}*\frac{dD}{dt}=1-D$ and *D* constrained to be ≤1. After each stimulus, *D* was multiplied by a constant *d* (≤1) representing the amount of depression per pre-synaptic AP, and updated as D→D*d. Between stimuli, *D* recovered exponentially back toward 1. We modeled depression using two factors *d*_1_ and *d*_2_ with *d*1 being fast and *d*_2_ being slow subtypes, and d=d1*d2. The parameters for modeling short-term plasticity are listed in Table 4. Our model did not have long-term synaptic plasticity.

**Table 4.** Parameters related to short-term presynaptic plasticity

		Parameters		
Connection	Short-term dynamics	D (maximum limit)	d_1_/d_2_	*τ*_D1_/*τ*_D2_ (ms)
FSI-PN	Depression	0.6	0.9/0.95	40 / 70
PN-PN	Depression	0.5	0.9/0.95	40 / 70
PN-FSI	Depression	0.7	0.9/0.95	40 / 70

#### Intrinsic connections

Except for FSI→FSI connections with both electrical and chemical synapses, all other connections were implemented as chemical synapses. Connection probabilities have been found to be distance-dependent for PN→PN contacts in BL, and we used 3%, 2%, 1%, and 0.5% probabilities for intersoma distances of <50, 50 − 100, 100 − 200, and 200 − 600 µm, respectively (Abatis et al., 2017). For connections involving interneurons, we used data from *in vitro* BL reports (Woodruff and Sah, 2007), with connections limited to pairs within ∼300 µm of each other. Probabilities in the model for unidirectional connections from FSI→PN were 34% and PN→FSI were 12%. Reciprocal connections between PNs and FSIs were set to 16%. Electrical connections between FSIs were set to 8%. When a pair of FSIs were electrically coupled, they had a 50% probability of a unidirectional chemical synapse, or a 25% probability of bidirectional synaptic connectivity. FSI pairs not electrically coupled had a 19% probability of unidirectional connectivity, and a 3% probability of bidirectional probability. These connectivity numbers in our model resulted in an overall synaptic FSI→FSI connectivity of 26%, of which 20% was unidirectional and 3% bidirectional. These probabilities resulted in the following intrinsic connectivity in the model: each PN received 24.98 ± 9.5 (mean ± SD, throughout the paper unless otherwise indicated) excitatory connections from other PNs, and 42.6 ± 12.9 inhibitory connections from FSIs; each FSI received 214.8 ± 58 excitatory connections from PNs, and 21.6 ± 7.4 inhibitory connections from other FSIs. Axonal conduction delay on all connections were distance dependent. See Tables 5, 6 for details.

**Table 5.** Connection probabilities, PN-PN connections

Type Parameters	PN to PN			
Connection range (µm)	<=50	[50,100]	[100,200]	[200,600]
Connectivity	3%	2%	1%	0.5%

Connectivity data are from Abatis et al. (2017). For all chemical synaptic connections, we designed axonal conduction delay to be $Del=\frac{Dis}{v}+mini_{del}+fluc+dt$, where *Del* denotes calculated conduction delay (ms), *Dis* is the intersoma distance (*µm*), *v* denotes conduction velocity (mm/ms, 1 mm/ms was used in this study), *mini_del_*denotes minimal conduction delay (*mini_del_*= 0.8 ms was used in this study), *fluc* denotes random fluctuation of conduction delay [ms, fluctuation of uniformly distribution of (−0.1,0.1) ms was used in this study], and *dt* denotes simulation time step, with *dt* = 0.05 ms was used in this study.

**Table 6.** Connection probabilities, FSI-FSI and PN-FSI connections

TypeParameters	Gap Junction between FSIs	FSI to FSI				Unidirectional FSI to PN	Unidirectional PN to FSI	Reciprocal PN to FSI
Overall connectivity	8%	26%				34%	12%	16%
Connectivity of subtype	--	Unidirectional		Bi-directional		--	--	--
		Between coupled FSIs	Between uncoupled FSIs	Between coupled FSIs	Between uncoupled FSIs	--		
		50%	19%	25%	3%	--		

Data is from *in vitro* BLA reports (Woodruff and Sah, 2007) limiting connectivity from/to FSIs to within ∼300 μm (also, see Cammarota et al., 2013).

#### BL afferents

PFC, PR, and ER strongly project to BL (McDonald and Mascagni, 1997; Shi and Cassell, 1999; Vertes, 2004), so we tailored our extrinsic inputs to match their spiking. To this end, we created surrogate spiking activity derived from putative PNs simultaneously recorded from the PFC, PR, and ER *in vivo* during the QW state (Headley et al., 2015). To do this, we created multiple surrogate spike trains where each was a random combination of the temporal firing rate profile of one neuron and the mean firing rate of another. The firing rate profile was the instantaneous firing rate of each unit, which was calculated by taking the inverse of the interspike interval in 1-ms steps. The instantaneous firing rate series was rescaled to have a mean rate of 1 Hz. Mean firing rate was the number of spikes dividing by the recording duration. A new spike train was created by multiplying one of the rescaled instantaneous rate series by a mean firing rate randomly drawn from the same population of units, and then passing each time step to a Poisson random number generator. When repeated multiple times, this creates an ensemble of spike trains that matches the distribution of firing rates, autocorrelations, and cross-correlations from the originally recorded ensemble. We generated an ensemble of 1800 Poisson spike trains to represent the activities of cortical regions upstream of BL, and each of these projected to a random 40 PNs, resulting in ∼95% of the PNs receiving at least one extrinsic input. The same extrinsic input also connected to FSIs that were within 300 µm of these 40 PNs with a probability of 0.3%; this resulted in each extrinsic input connecting to an average of 6 FSIs. Thus, PNs and FSIs (Hübner et al., 2014) received 2.97 ± 1.7 and 3.4 ± 1.9 extrinsic inputs, respectively.

To reproduce membrane potential fluctuation seen *in vivo*, we used a point conductance model that mimics stochastic background synaptic activity using an Ornstein–Uhlenbeck process (Destexhe et al., 2001). Specifically, stochastic background input I_bck_ had two independent components, excitatory and inhibitory, for both PNs and FSIs, modeled as follows:

(7) $$I_{bck}=g_{e}\left(t\right)\left(V-E_{e}\right)+g_{i}\left(t\right)\left(V-E_{i}\right)$$

where $g_{e}\left(t\right)$ and $g_{i}\left(t\right)$ are time-dependent excitatory and inhibitory conductances, respectively; $E_{e}=0mV$ and $E_{i}=-75mV$ are respective reverse potentials. The two are modeled as Ornstein–Uhlenbeck processes described below:

(8a) $$\frac{dg_{e}\left(t\right)}{dt}=-\frac{1}{\tau_{e}}\left[g_{e}\left(t\right)-g_{e0}\right]+\sqrt{D_{e}}\chi_{1}\left(t\right)$$

(8b) $$\frac{dg_{i}\left(t\right)}{dt}=-\frac{1}{\tau_{i}}\left[g_{i}\left(t\right)-g_{i0}\right]+\sqrt{D_{i}}\chi_{2}\left(t\right)$$

where $g_{e0}$ and $g_{i0}$ are average conductances, $\tau_{e}$ and $\tau_{i}$ are time constants, $D_{e}$ and $D_{i}$ are noise “diffusion” coefficients, $\chi_{1}\left(t\right)$ and $\chi_{2}\left(t\right)$are Gaussian white noise of zero mean and unit SD (for these parameter values, see Table 7).

**Table 7.** Parameters related to point-conductance model

Parameters	Excitatory source				Inhibitory source			
Neuron type	$g_{e0}$(nS)	$\partial_{e}$(nS)	$\tau_{e}(ms)$	$E_{e}$(mV)	$g_{i0}$(nS)	$\partial_{i}$(nS)	$\tau_{i}(ms)$	$E_{i}$(mV)
For PN	3.2	3	2.728	0	21	8	10.49	−75
For FSI	1.2	0.1	2.728	0	5.7	2.6	10.49	−75

The above two stochastic differential equations can be numerically modeled by using the following update rule:

(9a) $$g_{e}\left(t+\Delta t\right)=g_{e0}+\left[g_{e}\left(t\right)-g_{e0}\right]exp(-\Delta t/\tau_{e})+A_{e}N_{1}\left(0,1\right)$$

(9b) $$g_{i}\left(t+\Delta t\right)=g_{i0}+\left[g_{i}\left(t\right)-g_{i0}\right]exp(-\Delta t/\tau_{i})+A_{i}N_{2}\left(0,1\right)$$

where $N_{1}\left(0,1\right)$ and $N_{2}\left(0,1\right)$ are normal random numbers. $A_{e}$ and $A_{i}$ are amplitude coefficients with $A_{e}=\sqrt{\partial_{e}^{2}\left[1-exp(-2\Delta t/\tau_{e})\right]}$ and $A_{i}=\sqrt{\partial_{i}^{2}\left[1-exp(-2\Delta t/\tau_{i})\right]}$

Table 7 lists the parameters used in the point-conductance model. For each neuron, the excitatory and inhibitory conductances were, respectively, 3.2 ± 3 and 21 ± 8 nS for PNs and 1.2 ± 0.1 and 5.7 ± 2.6 nS for FSIs. However, the background inputs to FSIs was weaker to ensure FSIs spike with input from PNs, but not with solely background inputs (Börgers and Kopell, 2003; Economo and White, 2012; Lee and Jones, 2013).

#### Calculation of LFP

Gamma rhythms are detected by extracellular recordings of LFPs within the brain. In contrast, biophysical models of neuronal networks have typically detected gamma rhythms using other measures, such as network spiking rates (Brunel and Hakim, 1999; Brunel and Wang, 2003; Börgers et al., 2005; Economo and White, 2012; Chalk et al., 2016; Hoseini and Wessel, 2016; Palmigiano et al., 2017) or membrane voltages (Traub et al., 2000; Bathellier et al., 2006; Neymotin et al., 2011), both of which are only indirectly linked to the LFP (Buzsáki et al., 2012; Schomburg et al., 2012). The present study modeled LFPs using a first principles approach.

We first recorded transmembrane ionic currents from each compartment of the model cells using the extracellular mechanism in NEURON (Carnevale and Hines, 2005; Parasuram et al., 2016). The extracellular potential arising from each neuronal compartment was then calculated using the line source approximation method, which provides a better approximation than point sources (Gold et al., 2006; Schomburg et al., 2012). The extracellular potential of a line compartment was estimated as

(10) $$\emptyset_{EP}=\frac{I}{4\pi \sigma \Delta s}log|\frac{\sqrt{h^{2}+r^{2}}-h}{\sqrt{l^{2}+r^{2}}-l}$$

where, *I* denotes the transmembrane current from just that compartment, *Δs* the length of the line compartment, *r* the radial distance from the line, *h* the longitudinal distance from the end of the line, and l=Δs+h the distance from the start of the line (Holt, 1998; Parasuram et al., 2016). We chose a conductivity σ of the extracellular medium to be 0.3S/m (Goto et al., 2010; Einevoll et al., 2013). These individual extracellular potentials were summed linearly (Lindén et al., 2013) at 1-ms resolution, to obtain the LFP $\emptyset_{LFPs}$ for an *N*-neuron network with *n*-compartment-cells using the equation

(11) $$\emptyset_{LFPs}=\sum_{N=1}^{N\_neurons}\sum_{i=1}^{n\_source}\frac{I_{Ni}}{4\pi \sigma \Delta s_{N_{i}}}log|\frac{\sqrt{{h_{N_{i}}}^{2}+{r_{N_{i}}}^{2}}-h_{N_{i}}}{\sqrt{{l_{N_{i}}}^{2}+{r_{N_{i}}}^{2}}-l_{N_{i}}}|$$

where *N_i_* denotes *i* ^th^ compartment of *N* ^th^ neuron in the network. All 27,000 neurons contributed to the LFP in our study, different from previous studies (Bezaire et al., 2016) where only a subset of neurons did. This permitted investigation of both the individual contribution and correlation of all modeled neurons to the LFP at any of the electrode locations.

Since our model spans the entire spatial extent of BL, but only has a fraction of the total number of neurons, the density of neurons in our model is lower than the *in vivo* case. This means that neurons will, on average, be farther from the simulated LFP electrode than would occur in the actual BL. To correct for this, we rescaled the LFP by a correction factor that was estimated following Lindén et al. (2011), who suggest that LFP scales as the square root of the number of neurons, *N*, for uncorrelated synaptic inputs. However, that scaling factor was derived from a network of pyramidal neurons positioned in a disk with uniformly oriented dendrites, and was not for density per se but actually the radius of the disk with density held constant. To determine the scaling factor in our model, we systematically varied the number of cells used to calculate the LFP from a full model run. Varying the cell count from 1000 up to 27,000, we found that the SD of the LFP scaled with density following N^0.67^. Since cell density in rats is reported to range from 2.5 × 10^4^ to 2 × 10^5^/mm^3^ (Tuunanen and Pitkänen, 2000; Salm et al., 2004; Pêgo et al., 2008; Rubinow and Juraska, 2009; Chareyron et al., 2011), while the model density is 9840 neurons/mm^3^ (27,000 neurons, 1.4 × 1.4 × 1.4 mm), the LFP correction factor would correspondingly range from 1.9 to 7.5 [$({2.5\times 10^{4}/9.84\times 10^{3})}^{0.67}$ to $({2\times 10^{5}/9.84\times 10^{3})}^{0.67}$]. We chose the average of this range, 4.7, to scale the model LFP.

#### Multiple extracellular electrodes

To investigate the spatial propagation of model gamma we used a 9 × 9 × 9 grid of LFP electrodes evenly spaced throughout the network. Electrode sites were spaced at 125-µm intervals and were at least 200 µm away from the edges of the model. The LFP on each electrode was computed the same as in Equation 11.

### Model experiments and statistics

#### Spectral and cross-correlation analyses

Unless otherwise indicated, spectral decompositions were performed using Morlet wavelets ranging from 1 to 256 Hz in quarter octave steps. The width of the wavelet was seven cycles. To measure the amplitude at a particular frequency and time, we took the absolute value of the complex valued frequency domain representation of the signal. Phase was measured as the angle of the frequency domain representation.

The distribution of gamma burst properties were compared between the model and *in vivo* cases in Figure 2*F*. For both cases, wavelet power spectrograms were calculated and the mid-gamma band (64 Hz) was isolated. Peaks were detected and segmented in the time series of the mid-gamma power using a watershed function. To prevent spurious detections of peaks, before passing the data to the watershed function, we discretized gamma power into bins of half the average change in power between cycles. Once burst peaks were detected, we extracted their peak power (before discretization) and duration. Peak powers were transformed into percentile ranks and binned in steps of five percentiles, while durations were binned in steps of 5 ms.

For the single neuron resonance analyses (Fig. 2*G*,*H*), we calculated spectrums using the Welch Periodogram method (*pwelch* in MATLAB). The Hamming window taper size was 500 ms, in steps of 250 ms.

For the coherence spectrum between PN and FSI spiking (Fig. 7*D*), the cross-correlation and autocorrelation between these spike trains were calculated with 1-ms resolution. Then, they were converted to the frequency domain using the fast Fourier transform (FFT) and used to calculate coherence:

(12) $$Coherence=\left|\frac{{XCorr}_{PNandFSI}}{\sqrt{{ACorr}_{PN}{ACorr}_{FSI}}}\right|$$

Since both the PNs and FSIs exhibited gamma periodicity in their autocorrelation function, it was desirable to correct for this when estimating their cross-correlation. To do this, one can take the inverse FFT of the coherence spectrum, which yields the cross-correlation function in the time domain, but with periodicities arising from the autocorrelations factored out:

(13) $${CorrectedXCorr}_{PNandFSI}=invFFT\left(\frac{{XCorr}_{PNandFSI}}{\sqrt{{ACorr}_{PN}{ACorr}_{FSI}}}\right)$$

##### Entrainment to LFPs

For calculating the entrainment and preferred phase of cells, we first bandpass filtered the LFPs in the frequency band of interest using a two-pole Butterworth filter implemented with the MATLAB function *filtfilt*, which performs forward and backward filtering to minimize phase distortion. A Hilbert transform of the resulting signal was then computed to determine the phase and amplitude at each instant (Amir et al., 2018). This was used to assign a phase to each spike from a neuron. Entrainment of spiking to the LFP was computed as the mean resultant vector length using the Circular Statistics toolbox in MATLAB (Berens, 2009). This approach matched that in Amir et al. (2018). For our analysis of entrainment when disconnecting specific connections (see Results, Circuit elements critical for gamma genesis), we instead used the pairwise phase consistency (PPC) measure, which is insensitive to changes in spike count arising from alterations in firing rate (Vinck et al., 2010).

When computing correlation between distance and entrainment (Fig. 3*D*), cells were selected only within 100 µm from the four longest diagonals within the modeled cuboid structure, to minimize edge effects. To directly compare the decay in entrainment between PNs and FSIs (Fig. 3*D*), we fit a generalized linear model with a log link function and factors distance, cell type, and their interaction.

#### Detection of gamma bursts

For gamma burst extraction, we first applied zero-phase 60 − 80Hz Butterworth bandpass filter of order 4, followed by the Hilbert transform to obtain the analytic signal of the LFP (Dvorak and Fenton, 2014). Instantaneous gamma amplitude was the modulus of the analytic signal. Gamma burst occurrence was detected when the amplitude exceeded its mean by 2 SDs. Burst duration was defined as the duration during which the amplitude was >25% of the peak value for the burst.

#### Propagation of gamma bursts

Using the 9 × 9 × 9 grid of LFP recording sites (see Multiple extracellular electrodes), we examined the properties of gamma bursts as spatiotemporal events. Near the edges of our model, we found that the gamma bump in the power spectrum shrunk, likely due to the decrease in the number of neurons contributing to the LFP and decrease in the absolute number of connections between PNs and FSIs. Therefore, to compensate, we multiplied the Z-score of the LFP amplitude from each recording site by a correction factor *F* with the form:

(14) $$F=\sqrt{\frac{{GammaAmp}_{j}}{{TotalAmp}_{j}}}/\sqrt{\frac{{GammaAmp}_{middle}}{{TotalAmp}_{middle}}}$$

where GammaAmp is the area under the curve for the bump in the gamma band after removing a power law fit (Neske and Connors, 2016), TotalAmp is the total area under the curve for the power spectrum, *j* denotes the recording electrode, and *middle* indicates the value for the electrode in the center of the model. The Z-scored LFP Hilbert transformed amplitude at each site was multiplied by this factor.

We then identified gamma bursts as spatiotemporal events using a four-dimensional watershed algorithm that identifies contiguous regions of elevated gamma power that are convex. To minimize spurious detections of gamma bursts driven by small variations in the power, we discretized the Z-scored LFP Hilbert transformed amplitude into bins of 0.1. In order for a region to count as a gamma burst, its peak amplitude had to exceed two Z-scores. The borders of the burst region were delineated when power dropped by 25% of the peak. These were criteria were intentionally similar to the ones used for detecting gamma bursts on single electrodes (see above, Detection of gamma bursts).

After identification of a gamma burst, its properties were defined as follows. Gamma burst peak (Fig. 6*B1*) was defined as the maximum Z-scored LFP amplitude within the burst. Its time duration (Fig. 6*B2*) was calculated as the time duration during which at least one recording site within the watershed region was above the 0.25% of the peak Z-score. Gamma burst volume (Fig. 6*B4*) was defined as the number of recording sites that had reached the burst threshold with the same burst region.

To estimate propagation of gamma bursts, we identified the gamma region composed of electrodes with the same watershed label at each time step. Then, a burst center point was defined as the mean coordinate from all electrodes belonging to the same burst region. Gamma burst path length (Fig. 6*B5*) was calculated as the total distance that a burst center could travel in space.

To quantify instantaneous gamma burst synchrony (Fig. 6*B3*), we calculated the phase locking value (PLV) by using the unbiased PLV (Aydore et al., 2013) of 27 electrodes within a 3 × 3 × 3 grid located at the gamma burst peak.

#### Statistical analysis

Values are mean ± SD unless otherwise stated. Adjusted *R* ^2^ value were reported when data points were fit by a curve in Figure 3*D*,*E*. For Figure 5*E*, ANOVAs were performed on fitted general linear models and *p* values were reported. Two-tailed rank-sum tests were performed and *p* values were reported for Figure 7*B*,*D*. Watson–Williams tests by using Circular Statistics toolbox in MATLAB (Berens, 2009) were performed and *p* values were reported for Figure 7*C*. ANCOVA was tested and *p* values were reported for Figure 7*F*.

Original NEURON code for the model is available in ModelDB public database with access number 247968 (http://senselab.med.yale.edu/ModelDB/).

## Results

### Validation of the BL model

A biophysically realistic computational model of the rat BL was developed based on the known anatomy and cellular neurophysiology (for details and supporting empirical references, see Materials and Methods). The model was a cube with PNs and FSIs distributed randomly throughout (Fig. 1*A*,*B*). The dendritic compartments for both PNs and FSIs were oriented randomly and spanned several hundred microns from the cell body (Fig. 1*C*). Using this model, we tracked both the intracellular and extracellular signals associated with network events (Fig. 1*D1*). The model network included extrinsic afferents contacting both PNs and FSIs and intrinsic connections (Fig. 1*D2*), in keeping with previous anatomic (Paré et al., 1995; Muller et al., 2006) and physiologic (Washburn and Moises, 1992; Rainnie et al., 1993; Woodruff and Sah, 2007) observations.

Two types of PNs with marked (PN_A_) or negligible (PN_C_) spike frequency accommodation were used based on previous reports and our own investigations (see Materials and Methods; Fig. 1*E*,*F*). Our *in vitro* recordings revealed 41/56 PN_A_ and 15/56 PN_C_s, so our model used similar proportions (71% PN_A_, 29% PN_C_). We also included FSIs, which are the prevalent type in BL (McDonald and Mascagni, 2001; Fishell and Rudy, 2011), well characterized biophysically and synaptically (Rainnie et al., 2006; Woodruff and Sah, 2007), and are important for gamma genesis in cortical circuits (Bartos et al., 2007). Point conductance noise levels were adjusted so that the firing rates of PNs and FSIs matched those observed *in vivo* (Fig. 1*G*).

The LFP was calculated using the relative location of cellular compartments, their transmembrane currents, and an estimate of the extracellular conductivity (see Materials and Methods). The model LFP exhibited several well-established aspects of real extracellularly recorded APs and excitatory postsynaptic potentials (EPs; Fig. 1*H*,*I*). PNs produced stronger dipoles than FSIs, resulting in greater extracellular potentials. Near the soma, the simulated extracellular AP was negative going, larger for PNs, and faster for FSIs (Fig. 1*J1*,*2*, note scale difference). A unitary EPSC evoked in a dendritic branch produced an extracellular negativity near the dendrite that was substantially smaller than that arising from an AP (Fig. 1*J2*). Additionally, the amplitude of extracellular APs decreased with distance from the soma similarly between the model and *in vivo* cases (Fig. 1*H*,*K*).

### Emergence of gamma oscillations in the BL model

We next examined whether the spontaneous LFP at the center of our BL model (Fig. 2*A*) matched *in vivo* BL recordings when rats were in a QW state. A characteristic of the LFP *in vivo* is that its power spectrum can be fit by a 1/f^a^ power law (Buzsáki et al., 2012). BL recordings from Long–Evans rats showed a spectrum with the 1/f^a^ falloff, but also a bump in the gamma band between 54 and 70 Hz (Fig. 2*B*, gray lines). A gamma band signal was present in the model’s LFP, with similar power (101 mV^2^/Hz) and peak frequency (64 Hz; Fig. 2*B*,*C*, red line). However, the model’s spectrum had substantially less power below 30 Hz. This may reflect the absence of volume conducted activity or a lack of interactions with extrinsic structures. The former possibility was studied by Łęski et al. (2013), who showed that lower-frequency LFP components have a larger spatial reach and extended further outside the active population than high-frequency components. It has also been found that increasing the correlations between extrinsic afferents boosts power in the low-frequency band (Łęski et al., 2013; Hagen et al., 2016). Our model likely underestimates the contribution of correlated extrinsic inputs since our naturalistic spike trains only represented a fraction of the structures projecting to the BL, each presumably with their own correlated spiking. Consistent with this, we found that replacing the naturalistic correlated spiking processes of our extrinsic afferents with rate matched independent Poisson processes further reduced the power of low frequencies (Fig. 2*B*, green line). Importantly, gamma power from 50 to 80 Hz was unaffected by this change, supporting the hypothesis that it is locally generated and not imposed by upstream structures or volume conducted from adjacent regions. In addition, the gamma oscillations in our model occurred as intermittent bursts, like those found in BL *in vivo* (Fig. 2*D*,*E*). The probability distribution of gamma bursts with respect to their duration and amplitude was similar between the model and those recorded *in vivo* (Fig. 2*F*).

Neither the resonance properties of single cells (Fig. 2*G1–3*), nor the spectral content in EPSCs and IPSCs (Fig. 2*H1*,*2*) contributed to the gamma in the model. Also, there was no enhancement in the gamma-band when synaptic potentials were driven as Poisson processes with rates ranging from 1 to 200 Hz (Fig. 2*H3*). This leaves interactions between PNs and FSIs as the most likely candidate causing gamma.

### Entrainment of neurons to gamma in the LFP

We examined whether BL neurons showed a similar entrainment (Fig. 3*A*) to *in vivo* recordings (Amir et al., 2018). For neurons within 300 µm of the point where we calculated the LFP we measured their resultant vector (Fig. 3*B*,*C*). Both types of PNs were entrained to the gamma rhythm, spiking during the trough, with a mean resultant vector of 0.19 ± 0.07 (mean ± SD) for PN_A_s, and 0.17 ± 0.06 for PN_C_s. On the other hand, FSIs tended to fire during the ascending phase, and exhibited similar mean entrainment, 0.17 ± 0.04.

PNs and FSIs exhibited decreased entrainment to gamma at longer distances (Fig. 3*D*; PN: 50% falloff at 409 µm, adjusted *R* ^2^ = 0.37, df = 350; FSI: 50% falloff at 355 µm, adjusted *R* ^2^ = 0.67, df = 146). A generalized linear model also indicated a distance-dependent falloff (*t* statistic = −15.29, df = 496, *p* = 1.63 × 10^−43^) and no difference in falloff between cell types (distance × FSI, *t* statistic = −0.95, df = 496, *p* = 0.34). To rule out the possibility that the decrease in entrainment was driven purely by the distance-dependent decay in a neuron’s contribution to the extracellular potential, we examined the contributions of individual PNs and FSIs at different distances (Fig. 3*E*). Arguing against this, the falloff in the contribution of both PNs and FSIs to the extracellular potential was much sharper than the decay in their entrainment (PN: 50% falloff at 125 µm, adjusted *R* ^2^ = 0.59, df = 1435; FSI: 50% falloff at 113 µm, adjusted *R* ^2^ = 0.67, df = 749).

### Circuit elements critical for gamma genesis

To identify the circuit elements that support the generation of gamma oscillations, we systematically eliminated each connection type one at a time. Since the firing rates of PNs and FSIs could change dramatically in the disconnected models, we adjusted the point conductance noise of both cell types to bring them back into agreement with the original unperturbed model. For each perturbed model, we measured the power spectrum of the simulated LFP and unit entrainment to frequencies ranging from 1 to 256 Hz with the PPC measure, which is unbiased for changes in spike count (Vinck et al., 2010). To clarify the changes, we saw in the power spectrum, we calculated the cross-correlation between bursts of PN activity (time points where the firing rate of the PN population exceeded the 75th percentile) and spiking in the FSI population.

Removal of the FSI→PN connection eliminated the mid-γ bump in the LFP (Fig. 4*A1*), but increased power at frequencies below 20 Hz. Consistent with this, the entrainment of PNs by low frequencies was increased as well (Fig. 4*A2*), with a particularly prominent peak at 6 Hz (Fig. 4*A2*, inset). Also mirroring the LFP, PN and FSI entrainment to gamma was substantially reduced. This loss was evident in the response of the FSI network to bursts of PN activity, which now lacked a prominent gamma periodicity (Fig. 4*A3*). Instead, FSIs exhibited a slow undulation in firing that was capped with a transient burst of spikes in response to PN firing.

PN→FSI connections were also critical to gamma generation (Fig. 4*B1*). Their removal reduced the entrainment of PNs and FSIs in the gamma frequency range (Fig. 4*B2*). Compared to the previous model, the increase in power in the low-frequency band was smaller, so too was entrainment by PNs (Fig. 4*B1*,*2*). FSIs, on the other hand, were not entrained to the low-frequency rhythm, suggesting that it originated with the PN network, from which it was now disconnected. As expected, FSIs no longer responded to bursts of PN activity (Fig. 4*B3*).

The elimination of gamma by removing either PN→FSI or FSI→PN connections is in agreement with a PING based mechanism for gamma genesis (Whittington et al., 2000). This does not rule out the importance of connections within the PN and FSI populations. Since these connections were also present in the model, we also examined their contribution.

An interconnected network of FSIs that receives tonic excitatory drive can produce gamma oscillations, so called interneuron network gamma (ING; Whittington et al., 2000), which may occur in the model. Eliminating FSI→FSI connections (including gap-junctions), shifted the peak frequency to 23 Hz (Fig. 4*C1*). Likewise, both PNs and FSIs shifted their maximal gamma entrainment to the new peak frequency (Fig. 4*C2*). Since the timescale of feedback inhibition affects the frequency of gamma (Wang and Buzsáki, 1996), the lower frequency likely reflects the increased duration of the PN-evoked FSI burst, which arose from the removal of feedback inhibition generated by FSIs onto themselves (Fig. 4*C3*).

Removing PN→PN connections had a modest effect on gamma generation, shifting the peak frequency slightly lower, which was also reflected in the entrainment of PNs to gamma (Fig. 4*D1*,*2*). Notably, there was a large drop in spectral power at lower frequencies, suggesting that it partly depends on recurrent activity within the PN network. Befitting these minor changes, FSI responses to PN bursts were only marginally affected (Fig. 4*D3*).

Altogether these results suggest that reciprocal interactions between PNs and FSIs support the generation of gamma oscillations in BL, and that their frequency is affected by connections within the PN and FSI populations.

### Synaptic basis of gamma coordination in single cells

Since gamma in our model was similar to the *in vivo* case, we explored how the convergence of excitatory and inhibitory synapses affects a neuron’s entrainment to gamma. We chose 300 PNs and FSIs at random and recorded their EPSCs, IPSCs, spike times, and surrounding LFP (sampled ∼200 µm away; Fig. 5*A*). We limited ourselves to neurons that fired over 100 spikes in the 165 s of simulation time, so only PN_c_s were present in the PN sample.

Given that synaptic inputs constitute a major determinant of neuronal firing, we first assessed the spectral content of the EPSCs and IPSCs impinging on PNs and FSIs (Fig. 5*B*). On average, IPSCs exhibited a pronounced bump in the mid-gamma band for both cell types. This contrasted with EPSCs, which lacked increased gamma-band power for PNs, and only exhibited a shallow increase in FSIs. The presence of a gamma bump in both synaptic current power spectra for FSIs, but not PNs, suggested that the coherence between excitatory and inhibitory synaptic currents differs between cell types. Indeed, EPSC-IPSC coherence at the gamma frequency was higher in FSIs than in PNs (Fig. 5*C*).

To determine whether this effect was related to spiking during gamma (Fig. 5*D*), we calculated the mean EPSCs (Fig. 5*D1*, red lines) and IPSCs (Fig. 5*D2*, blue) during gamma cycles with (solid) and without spikes (dashed), and their difference (Fig. 5*D3*). For PNs, spiking was associated with an increase in EPSCs, which started rising during the latter half of the prior gamma cycle, and peaked at the trough of the oscillation, when PNs normally fire (Fig. 3*C*). As was evident in the power spectrum for the PN EPSC current (Fig. 5*B*, left, red), no gamma periodicity was evident in the EPSC trace. On the other hand, the IPSCs tracked the ongoing LFP gamma rhythm (Fig. 5*B*, left, blue). While there was a slight decrease in IPSC strength during the first half of the gamma cycles containing spikes, this reversed itself during the latter half, with inhibition increasing following the phase when PN spiking normally occurs (Fig. 3*C*), likely reflecting feedforward activation of the FSI network.

FSIs exhibited a different pattern of postsynaptic currents during gamma cycles in which they spiked (Fig. 5*D*, right). The EPSCs were entrained to the ongoing gamma rhythm and elevated just before the neuron spiked, during the ascending phase of the gamma cycle. Also, unlike PNs, FSIs showed a decrease in IPSC amplitude throughout the gamma cycle in which they spiked, followed by a return to baseline. One similarity between PNs and FSIs was that their IPSCs were similarly entrained to the gamma rhythm. These results suggest that PN spiking during a gamma cycle is mostly dependent on the presence or absence of an EPSC, while FSIs are more likely to spike when both EPSC amplitudes are increased and IPSCs are decreased. Despite these differences, it is worth noting that PNs and FSIs tended to spike during the phase of the gamma cycle when EPSCs were at their maximum and IPSCs were at their minimum. Our finding that spiking during gamma cycles was accompanied by both EPSCs and disinhibition of IPSCs supports the claim that the gamma in the BL network arises through a PING mechanism, rather than the recurrent-excitation-inhibition (REI) mechanism found in models of visual cortex (Chariker et al., 2018).

Since spiking during gamma depends on the pattern of postsynaptic currents, it could be that a neuron’s entrainment to gamma depends on how many excitatory and inhibitory synapses it receives. For each neuron, we measured their entrainment to gamma using the resultant vector metric and determined how it varied as a function of their number of excitatory and inhibitory afferents (Fig. 5*E*). For PNs and FSIs there was a significant positive relationship between entrainment and the number of inhibitory afferents received (PN: *F*_(1,296)_ = 35.8, *p* = 6.3 × 10^−9^; FSI: *F*_(1,296)_ = 770.3, *p* = 2.3 × 10^−84^). However, the number of excitatory afferents did not alter entrainment in PNs (*F*_(1,296)_ = 0.16, *p* = 0.69), but decreased it in FSIs (*F*_(1,296)_ = 264.8, *p* = 5.7 × 10^−43^). Notably, FSI entrainment was enhanced when both inhibitory and excitatory afferents increased in tandem (*F*_(1,296)_ = 5.1, *p* = 0.025). Overall, the dependence of PN and FSI entrainment on the number of inhibitory connections agrees with the relatively strong gamma periodicity of IPSCs.

### Spatiotemporal properties of gamma bursts

Gamma oscillations not only persist in time but extend across space. This introduces uncertainty about the amplitude and duration of gamma bursts when recording from single sites. This is not a problem for our model, which can support an arbitrary number of recording sites arranged in any desired configuration. Thus, we characterized gamma bursts as spatiotemporal phenomena.

A 9 × 9 × 9 grid of recording sites with an interelectrode spacing of 125µm spanned the entire BL model (Fig. 6*A*). Spatiotemporal gamma bursts were identified in a four-dimensional space (three spatial and one temporal). We measured gamma amplitude at each site and identified spatially contiguous regions of elevated gamma power at each time step. To count as a burst power had to peak above 2 SDs of the mean and the edges of the burst were thresholded at 25% of the peak power.

By surveying the entirety of each gamma burst, we could unambiguously measure their properties (Fig. 6*B*). Gamma burst peak amplitudes hugged the cutoff threshold we had set at two Z-scores, with a mean of 2.8, and <1% above 5 (Fig. 6*B1*). Burst durations were 108 ms on average, enough time to encompass several gamma cycles (Fig. 6*B2*). We also measured the mean phase locking of the LFP in the gamma band surrounding the location of peak power in the gamma burst. Most bursts had an average PLV of 0.87 (Fig. 6*B3*), indicating that at the center of the burst, adjacent LFP recording sites were highly phase coherent.

Tracking across multiple recording sites revealed the spatial properties of gamma bursts. Localized increases in gamma power could start in one part of the model, spread to adjacent areas, and then dissipate. Most bursts were local, encompassing 44.3 recording sites on average out of a possible 729 total (6% of the BL volume; Fig. 6*B4*). If the mean volume of a gamma burst were arranged as a sphere, it would have a radius of ∼270 µm. Despite being local, the center of the burst could move throughout the BL, with bursts on average traversing 472 µm from their start to end times (Fig. 6*B5*). Taking the distance a burst traveled, and dividing that by the burst duration, we could measure the average speed of its center, which was 4.4 µm/ms (Fig. 6*B6*), or 68 µm per gamma cycle (assuming a cycle duration of ∼15 ms).

These results highlight the fact that gamma bursts, even in relatively small nuclear structures like BL, can be highly localized. However, they are not spatially confined; they can emerge at one location and terminate in another. Such propagation may mediate interactions between distant cell populations.

### Computation in the BL network

To explore how the microcircuitry generating gamma affects the interaction between BL neurons, we randomly assigned PNs in the network to one of two populations (group 1 and group 2), each receiving a different set of extrinsic afferents (Fig. 7*A1*). The simulated LFP was recorded from the center of the model, along with the spiking activity, IPSCs, and EPSCs of nearby neurons. Each population had its extrinsic afferents driven as Poisson processes at a fixed rate (Fig. 7*A2–4*). For these simulations we used a reduced BL model limited to 1000 neurons, with similar connectivity rules and proportions of cells types as found in the full model.

We first examined how increases in afferent drive to group 1 PNs affected synchronous spiking within each group (Fig. 7*B1*). Randomly selected pairs of neurons had their spike cross-correlations calculated. A robust gamma frequency periodicity was evident in the cross-correlation function of both groups (Fig. 7*B2–4*), with the group receiving the strongest afferent drive exhibiting greater synchronous spiking [group 1 > group 2, rank-sum test z value = 11.8, *p* = 6.8 × 10^−32^ (Fig. 7*B2*); group 2 > group 1, z value = −13.7, *p* = 1.6 × 10^−42^ (Fig. 7*B4*)]. When both groups were driven with equal strength there was not significant difference in their spiking synchrony (group 1 = group 2, z value = 0.2, *p* = 0.81; Fig. 7*B3*).

Previous analyses revealed that EPSCs in FSIs had a gamma component (Fig. 5*B*,*D*), so we next determined whether the more strongly driven PN group (group 1) contributed to this effect. Spikes times were recorded in tandem with the phase of the mean EPSC current in the gamma band across all FSI neurons (Fig. 7*C1*). PNs tended to spike before the peak of the EPSC, with a quarter cycle lag (Fig. 7*C2–4*). Whichever group was driven strongly had greater entrainment to the FSI EPSCs [group 1 > group 2, group 1 spike resultant vector, RV = 0.54, group 2 RV = 0.5, Watson–Williams test, *p* = 1.1 × 10^−16^ (Fig. 7*C2*); group 2 > group 1, group 1 RV = 0.5, group 2 RV = 0.54, *p* = 4.1 × 10^−13^ (Fig. 7*C4*)]. When both groups were driven equally their entrainment with FSI EPSCs did not differ (group 1 = group 2, group 1 RV = 0.56, group 2 RV = 0.56, *p* = 0.17; Fig. 7*C3*).

Given that FSI spiking depends on EPSCs, it should be the case that their spiking will be controlled mostly by the PN population more entrained to the EPSC rhythm, i.e., the strongly driven group. We examined this by measuring the cross-correlation between PNs from each group and all spikes emitted by the FSI network (Fig. 7*D1*) after correcting for the autocorrelations inherent in both spike trains. This revealed a peak in the cross-correlation at a 4-ms lag (Fig. 7*D2*, inset), consistent with the previous analysis (Fig. 7*C2–4*). Casting the normalized cross-correlation function into the frequency domain returned a measure of coherence between PN and FSI spiking. This revealed that the more strongly driven PN group had higher coherence with the FSI population, particularly in the gamma band [group 1 > group 2, rank-sum test, z value = 2.6, *p* = 0.009 (Fig. 7*D2*); group 2 > group 1, rank-sum test, z value = −3.7, *p* = 2.3 × 10^−4^ (Fig. 7*D4*)], and there was no difference when both groups were equally driven (group 1 = group 2, z value = −0.3, *p* = 0.79; Fig. 7*D3*).

Since the more strongly activated PN group exerts a greater influence over the FSI network, it seemed likely that this would lead to suppression of the weaker PN ensemble. Indeed, we found that as group 2 was more strongly excited, it diminished the firing of group 1 (Fig. 7*E1*). Presumably, this effect was mediated by the PN→FSI connection, which were also critical for generating the gamma rhythm. Eliminating these connections reduced the difference in firing rates between groups 1 and 2 (Fig. 7*E2*), suggesting that the FSI network may mediate competition between PN ensembles.

If FSIs allow for competition between ensembles of PNs, and FSIs tend to project locally, one would expect that any competition effect will operate when the groups of PNs overlap spatially, sharing the same FSI network, and not when they are far apart from one another. To explore this possibility, we returned to the original full model with 27,000 neurons. Our spatial analyses of gamma bursts revealed that they spanned an average radius of ∼270 µm (Fig. 6*B4*), so we created spheroidal subgroups of PNs with a similar size that were driven by Poisson afferents. When those groups overlapped in the model, they exhibited the competition effect (Fig. 7*F1*). Increasing the afferent drive onto group 2 reduced the firing rate of group 1, whose drive was held constant. However, if they were completely separated then no competition was evident (for group 1 interaction between separation × group 2 afferent drive, ANCOVA, *F*_(1,3474)_ = 7.8, *p* = 0.005; Fig. 7*F2*). Also in agreement with the stronger group 1 exerting downward pressure, for group 2, we found a significant main effect increase in firing rate on spatial separation (*F*_(1,3471)_ = 120.5, *p* = 1.4 × 10^−27^).

The above analyses suggest that the competition between ensembles arose from the heightened gamma-band interaction between a strongly driven group of PNs and the local FSI network. Since the competition was reflected mainly as a suppression of spiking, it was probably mediated by IPSCs impinging on the PN network. Moreover, since PN spikes tended to precede FSI spikes (Fig. 7*D2* , inset), the dominant PN group should, on average, control the inhibitory rhythm, which would periodically modulate neuronal responsiveness to excitatory drive, as found in sensory cortices (Cardin et al., 2009; Ni et al., 2016). To examine this, we measured the relationship between EPSC strength and spiking probability during different gamma phases. For the weakly driven group, the trough phase of the gamma cycle showed the strongest relationship between EPSC strength and spiking probability, likely because of the waning inhibition (Fig. 7*G*, bottom left). Once that inhibition was reinstated during the peak, it shunted EPSCs and degraded their ability to drive spiking. We sought to determine if this dependence was specific to gamma generation. Removing the PN→FSI connections eliminates gamma in the network while sparing inhibitory drive onto PNs. Doing so diminished the gamma phase dependence of sensitivity to EPSCs (Fig. 7*G*, bottom right).

## Discussion

A biophysically and anatomically detailed 27,000-cell model of BL produced transient gamma oscillations with properties similar to those seen *in vivo*. This concordance extended to gamma band entrainment and preferred phase of PN and FSI spiking. BL gamma arose from reciprocal interactions between PNs and FSIs, which suggests a PING mechanism of rhythmogenesis. Having established the validity of our model, we explored questions that could not be tested experimentally. Underscoring the importance of inhibitory connectivity, both PNs and FSIs were more synchronized to gamma if they received a greater number of inhibitory afferents. Finally, this connectivity produced lateral inhibition, which mediated competition between ensembles of PNs, with the more strongly driven PN population exerting greater control over the FSI network. It is noted that computational models of transient gamma have not been reported for the amygdala, and the ones reported for other brain regions are typically not biophysically or anatomically detailed to the extent that we present here.

### Emulation of BL gamma

BL has similar cell types and connectivity as found in cortical circuits (Swanson, 2003). A majority of its neurons are composed of PNs and FSIs (Rainnie et al., 1993, 2006), are reciprocally connected with each other (Muller et al., 2005; Woodruff and Sah, 2007), and like cortex (Braitenberg and Schu¨z, 1991) the BL receives a substantial portion of its input from other cortical regions (Sah et al., 2003). Also as in cortex, examples abound of gamma oscillations recorded from the amygdala (Oya et al., 2002; Bauer et al., 2007; Stujenske et al., 2014), and *in vitro* slice preparations can be coaxed into generating gamma through pharmacological manipulations (Sinfield and Collins, 2006; Randall et al., 2011) similar to those used in cortical slices (Whittington et al., 2000). Thus, the importance of BL gamma oscillations seems assured, and yet our understanding of how BL produces gamma is limited.

As a first step, we examined a fundamental question: are the local circuits present in that area sufficient to produce the intermittent rhythm measured *in vivo*? Doing this required collating the anatomic and physiologic properties of BL neurons into a model and driving it with spike trains mirroring those found in upstream areas. Then, we calculated the extracellular field that could be picked up by recording electrodes (Pesaran et al., 2018). On running our simulations, the LFP exhibited a peak in the gamma band that was in the same range of frequencies and amplitudes as observed *in vivo*. Moreover, the spiking of both PNs and FSIs were entrained to the gamma rhythm as found *in vivo* (Amir et al., 2018). This concordance suggested that the BL network, as captured by our model, is sufficient for the generation of gamma like that seen *in vivo*.

However, the model exhibited far less power in the low-frequency bands. Given the concordance between our model of the extracellular field for both APs and activity in the gamma band, we suspect that this discrepancy reflects the extrinsic origins of low-frequency activity. One possible source is volume conduction from adjacent cortical regions. It may also be that afferent synapses from upstream areas contribute to the low-frequency band. Supporting this argument, driving the model with spike trains derived from cortical recordings increased power in the low-frequency band compared with rate matched Poisson inputs. Besides neocortical and paleocortical afferents, BLA neurons receive direct projections from the ventral hippocampus, and in turn respond to hippocampal theta (Bienvenu et al., 2012) and intermittent sharp wave associated population bursts (Girardeau et al., 2017), both of which occupy the low-frequency range.

### BL microcircuitry presented unique challenges to gamma rhythmogenesis

The generation of gamma oscillations in our BL model was not a foregone conclusion. While qualitatively the BL network contains the microcircuitry required for producing gamma oscillations, quantitative differences in the properties of this network from previous PING models may have precluded gamma generation.

For a PING rhythm to emerge PNs must fire in advance of FSIs (Whittington et al., 2000), and thus increasing the average firing rates of PNs contributes to the strength of gamma oscillations produced in PING models and in slices (Adesnik and Scanziani, 2010). PN firing rates in the BL are smaller than those in the cortical models used to simulate gamma by at least a factor of 6 (Börgers and Kopell, 2008; Hoseini and Wessel, 2016; Chariker et al., 2018), which may have diminished the network’s ability to produce a PING type gamma rhythm. Also important is the strength of inhibitory feedback in the network (Börgers et al., 2012). Two factors that contribute to this are the strength of connections between PNs and FSIs and the number of FSIs in the network. We and others have found that the BL has a lower proportion of interneurons (McDonald and Mascagni, 2001) compared with standard PING models (Börgers and Kopell, 2008; Hoseini and Wessel, 2016; Chariker et al., 2018). Moreover, the connection probability between PNs and FSIs in BL is lower (Woodruff and Sah, 2007) than the aforementioned models. Thus, a priori the BL network may not be capable of producing gamma, and so our model was essential to at least test the possibility theoretically.

Another difference between our BL model and cortical-based PING models is a lower proportion of recurrent connectivity among PNs. Brunel and Wang (2003) found that including recurrent connections between excitatory units in a PING model could downshift the frequency of the network oscillation by half. On the other hand, our BL PING model has fairly little dependence on these connections, since their elimination did not substantially affect the prominence or peak frequency of gamma (Fig. 4*D*). In addition, other models of gamma generation depend on the recurrent excitation among excitatory units. For instance, the REI model (Chariker et al., 2018) contains a network of reciprocally connected excitatory and inhibitory units. Unlike PING models, the gamma-periodic bursts of activity depend on the buildup of recurrent excitation in the PN network, which raises the membrane potential for all units in the network, and drives them to fire synchronously. This mechanism for producing gamma is distinctly not PING, since the excitatory and inhibitory units activate simultaneously, instead of with the inhibitory units following the excitatory. Moreover, since BL PNs and FSIs *in vivo* fire during different phases of the gamma cycle (Amir et al., 2018), it is likely that the REI mechanism does not operate within the BL.

Thus, our model establishes the sufficiency of the BL network to produce gamma oscillations, and for those to arise via a PING mechanism. Despite that, it was also possible that any gamma produced by the model would be of insufficient power to comprise what is observed in the LFP *in vivo*. To address this, our model included a realistic estimation of the LFP, which most PING models lack (Brunel and Hakim, 1999; Traub et al., 2000; Brunel and Wang, 2003; Börgers et al., 2005; Bathellier et al., 2006; Neymotin et al., 2011; Economo and White, 2012; Chalk et al., 2016; Hoseini and Wessel, 2016; Palmigiano et al., 2017; Chariker et al., 2018). Since our model LFP exhibited gamma oscillations that were close to the frequency and power of those recorded *in vivo*, it establishes the sufficiency of the local microcircuitry in BL to explain the gamma observed in LFP recordings *in vivo*.

### Circuit contributions to the properties of gamma oscillations

Cortical gamma oscillations are thought to arise from an interaction between PNs and FSIs, whereby PNs strongly drive a recurrently connected network of FSIs, that in turn inhibit PNs for a short period of time, after which PNs are able to restart the cycle over again. We determined whether a similar mechanism was operating in our BL simulations, and investigated qualitative and quantitative features of the underlying microcircuits. Systematically removing each class of connections revealed that either the connections from PNs to FSIs or vice versa were crucial for generating the gamma rhythm, which is in agreement with a PING type mechanism. Although interactions between FSIs are sufficient to produce a gamma rhythm (Whittington et al., 2000), this was not the case in our model. Instead, they affected the peak frequency. This could be functionally relevant because presynaptic receptors specific to synapses between interneurons (Cossart et al., 2001) may be able to regulate the frequency of gamma rhythms.

The dynamics of EPSCs and IPSCs in our model (Fig. 5*B*,*D*) mostly aligned with those from a recent *in vitro* model of cortical gamma (Salkoff et al., 2015). Using paired whole-cell patch clamp recordings, synaptic currents were recorded in one cell, and correlated with the spiking of an adjacent one. The power spectrum of IPSCs in PNs and FSIs exhibited a prominence in the low gamma band, while EPSCs in PNs did not. Moreover, they observed that IPSCs weakened before PN and FSI spiking, after which they rebounded. We observed that pattern for PNs, while for FSIs the IPSCs returned to baseline after spiking. Another significant difference was that they only observed substantial increases in EPSC amplitude surrounding spikes from FSIs, but not PNs. This likely resulted from the fact that whereas FSIs receive convergent inputs from many PNs (Oswald et al., 2009), excitatory synapses are sparsely shared between PNs; in all likelihood the afferents that elicited spiking in one PN did not collateralize onto another. In contrast, we could monitor both the EPSC and spiking in the same cell and found a robust EPSC increase during PN spiking.

One unknown aspect of gamma generation that cannot be readily explored at present either *in vivo* or *in vitro* is the contribution of a neuron’s particular complement of excitatory and inhibitory afferents to its entrainment by the gamma rhythm. In addressing this, we found that the number of inhibitory afferents was particularly important for how a neuron would be affected by gamma. PNs and FSIs with more inhibitory contacts were more strongly entrained to gamma, while only FSIs showed a significant reduction in entrainment as the number of excitatory afferents increased. In agreement with these results, a previous study from our group (Amir et al., 2018) found that PNs that received a monosynaptic input from a nearby FSI showed stronger entrainment to gamma. However, our model differed from this study regarding the contribution of excitatory connections onto FSIs. In Amir et al. (2018), the presence of an excitatory monosynaptic contact onto an FSI was associated with increased entrainment, whereas in our model, it was not. Perhaps resolving this disagreement, we found that a concomitant increase in both excitatory and inhibitory contacts onto an FSI raised entrainment beyond what was expected by either connection type alone. Thus, our two studies could be reconciled if BL FSIs that receive more afferents from PNs also receive more from other FSIs. Perhaps this suggests that inhibitory and excitatory afferents need to be tuned for single neurons to be strongly entrained by the gamma rhythm. This may be reflected by experience-dependent plasticity of inhibitory networks (Donato et al., 2013).

### Computations emerging from gamma generating circuits in BL

In cortical networks, several functions have been ascribed to gamma, but a particular emphasis has been placed on their ability to synchronize spiking (Tiesinga et al., 2008) and mediate competition between cell assemblies (Börgers et al., 2005; de Almeida et al., 2009; Palmigiano et al., 2017). Our model exhibited both of these phenomena. PNs synchronized their spiking by phase-locking to the gamma rhythm. This could be crucial to BL function since its neurons fire at very low rates, and so it is likely they need to coordinate their activity to drive robust postsynaptic integration. We also found that activating a subset of PNs in the network drove a suppression of those that received a weaker input, and that gamma genesis played a critical role in this effect. This competition may be important in the BL for the selective recruitment of particular PN ensembles, allowing it to drive its many downstream targets with greater specificity.

## Conclusions

Gamma oscillations are a feature of cortical processing that is also expressed in BL, where they are likely generated via similar mechanisms, and perhaps perform the same functions. Moving forward, there are two reasons why having a detailed biophysical model of gamma generation in BL is important. First, we need a better understanding of the variety of situations that bring about and alter gamma oscillations, and a concrete model provides a framework in which these observations can be integrated with one another. Second, a biophysically detailed model can serve as a testbed for exploring the means and algorithms that might be used to alter these oscillations (Witt et al., 2013), and the functions of the BL itself.

10.1523/ENEURO.0388-18.2018.sm1 Supplementary Material 1 Supplementary Code used in article. Download Supplementary Material 1, ZIP file

## Synthesis

Reviewing Editor: Upinder S. Bhalla, National Centre for Biological Sciences, Tata Institute of Fundamental Research; Decisions are customarily a result of the Reviewing Editor and the peer reviewers coming together and discussing their recommendations until a consensus is reached. When revisions are invited, a fact-based synthesis statement explaining their decision and outlining what is needed to prepare a revision will be listed below. The following reviewer(s) agreed to reveal their identity: Gaute Einevoll. Note: If this manuscript was transferred from JNeurosci and a decision was made to accept the manuscript without peer review, a brief statement to this effect will instead be what is listed below.

1. The reviewers agreed that the paper was interesting and technically

accomplished, with important findings.

2, The authors must present the anatomical organization of the modeled system,

including cellular geometries individually and with respect to the other cells

in the population.

3. The calculation for the LFP needs to be better justified in relation to

previous studies.

4. The novelty of the work should be better presented, and placed in context

of similar work especially pertaining to gamma in other brain areas.

5. The relationship between the reported gamma to other frequencies needs to

be better analyzed, both for bursts and entrainment.

6. The authors must provide their code as required by the

Journal policy: “Policy on Computer Code and Software

Computational models: New computational neuroscience computer code must be included with the initial submission as Extended Data to ensure reproducibility...”

They should really have done this at the first submission.

The detailed comments of the reviewers are appended as they provide more

details and bring up some useful additional points.

================================================================

Reviewer 1:

The paper reports a pioneering modeling study the a part of amygdala (basolateral complex). The model consists of 27000 neurons, modeled as 2-compartment or 3-compartment neurons, and interestingly and importantly the model predicts both spikes and local-field potentials (LFPs). I find the approach to be sound and the work very accomplished.

Comments to the Authors

The paper of Feng et al. reports a pioneering modeling study the a part of amygdala (basolateral complex). The model consists of 27000 neurons, modeled as 2-compartment or 3-compartment neurons, and interestingly and importantly the model predicts both spikes and local-field potentials (LFPs). I find the approach to be sound and the work very accomplished. Since I also find the questions addressed to be both important and timely, I am supportive of publishing of this work in eNeuro.

I have, however, some comments and questions that I would like the authors to address prior to publication:

1. I miss a figure of the anatomical organisation of the basolateral complex of

the amygdala. This organization, in particular on the geometric organisation of

the dendrites of the different neurons, could have a strong effect on the LFP.

2a) On l. 301 it is stated that the dendrites of the neurons are randomly oriented. Is this what is observed in the amygdala? (I believe that in other nuclei the dendrites could have a radial organisation.)

2b) If not, I would suggest that other geometrical configurations of the dendrites also should be considered. This may not only affect the overall amplitude of

the LFP, but also the shape of LFP power spectra. This follows from the observation that (i) correlated synaptic input combined with (ii) spatially aligned dendrites can give a boost of the LFP at low frequencies (see Leski et al, PLoS Comp Biol 2013)

2c) This could maybe contribute to an explanation for why the low-frequency powe of the LFP is smaller in the model than what is seen in experiments? (as mentioned on l. 564 and in the Discussion)

2d) Note that the lack of low-frequency power in predicted LFP was also observed

in models for cortical LFP, see Hagen et al, Cerebral Cortex (2016). It would be good if a connection to this previous study could be made.

3. On l. 424 it is assumed that the LFP is proportional to the density of neurons. Is there a justification for this? In, for example Linden et al, Neuron (2011), it was found that in some network regimes (low synaptic input correlation) the LFP scales like the square root of the density while with large synaptic input correlation the scaling was more linear.

Minor comments:

l. 71: anatomically detailed -&gt; anatomical realistic

l. 148: referral to “one dataset whose manuscript is in preparation”. This seems unorthodox.

l. 306-317: The used of “x” for product is unnecessary. I suggest to remove them throughout the manuscript.

l. 322: Must be stated that V is in units of mV (or whatever)

l. 332: The use of “*” for product s unnecessary. I suggest to remove them.

l. 389: Give formula for Ornstein-Uhlenbeck process

l. 454: once -&gt; one

l. 559: Can't anything be fit by a 1/f power law over a short frequency-range? What is meant?

l. 590: How can the entrainment of rates have anything to do with extracellular potentials in this model?

=============================================================

Reviewer 2

The authors constructed a biophysically and anatomically detailed model of the basolateral amygdala (BL), which reproduced several properties of the network observed experimentally. In particular, this model generated transient gamma oscillations, as reported by these authors previously. Using this model, they characterized spatial spread of gamma as well as network and synaptic properties.

I found their model comprehensive, the questions well motivated, and analysis thorough. I have some questions and comments though, as described below.

1. Given that there are several detailed ING-PING type models of gamma oscillations, the authors should specify in more detail which of their results are new in terms of modeling. I understand that parameters have been optimized for BL, which is a new thing, but some results, such as bursty nature of gamma, have been shown in other brain areas. For example, the bursty nature of gamma has been described by Bob Shapley's group, who mentioned gamma as “filtered noise”, see Burns et al., 2010 and 2011, JNS, Xing et al, 2012, JNS. Their earlier models were more abstract (based on variants of Wilson-Cowan), such as Kang et al., 2010, JCN, but more recently they also have detailed biophysical models (see work of Chariker et al., 2018, JNS). As mentioned by the authors themselves, several other groups have also used ING-PING type models to describe gamma. At the very least, the authors need to have a detailed Discussion about their model versus others, and in particular, if there is anything new that has not been shown previously in other parts of the brain.

2. Intermittent bursts of gamma (Figures 2 and 6): the model appears to have intermittent bursts of power at all frequencies, as seen in Figure 2E. Is there anything particular about the gamma band? If the authors really want to emphasize the properties of gamma bursts, they should compare gamma bursts with bursts at other frequencies. In this regard, recently it has been suggested that the bursty nature of gamma actually arises due to the variability in the spectral estimator (Subhash Chandran et al., 2018, JNP), and that previous studies have underestimated the burst duration. If the authors really want to emphasize the burst properties (Figure 6), they should ensure that their results do not suffer from signal processing artifacts (perhaps by changing the filter setting and testing if that changes the parameters) and also compare the burst statistics with nearby frequency bands (say between 40-60 and 80-100 Hz).

3. Gamma entrainment (Figure 3): I was surprised that the authors only focused on the gamma band instead of comparing the phase locking etc in the entire frequency range. For example, is the phase locking shown in Figures 3B and 3C unique to gamma band? What about the real data? The authors should show spike-field coherence or PPC for both real and model data here before dissecting the network and cellular mechanisms in later figures (in which some of these analyses are done).

4. Figure 5C shows an increase in coherence for the PN population at high frequencies. Any idea why that is the case?

5. Figure 7G - when the network does not produce gamma, the peaks/troughs etc have no meaning, right? So it is surprising that no difference in spike probability is observed?

Minor

1. Please label A1 and A2 in Figure 1.

2. Line 1307: “with” the effect, not “which”.

## Author Response

Dear Dr. Bhalla and Reviewers,

We appreciate the positive review. The concerns raised have been addressed fully, and the manuscript has been updated appropriately, as we detail in the point-to-point responses below, with the review comments and comments in bold and plain font type, respectively.

The process has helped improve the manuscript, and we thank all of you for the comments.

Sincerely,

F Feng, D Headley, A Amir, V Kanta, Z Chen, D Pare, S Nair

Synthesis Statement for Author (Required):

1. The reviewers agreed that the paper was interesting and technically accomplished, with important findings.

We appreciate the comment.

2, The authors must present the anatomical organization of the modeled system, including cellular geometries individually and with respect to the other cells in the population.

We have revised Figure 1 to reflect the large scale spatial organization of our BL model and its cell densities (Figure 1A,B). We also provided an example of the random spatial orientation of the dendritic processes for our model neurons (Figure 1C). This organization fits the reports of Golgi stain studies in the rat BL (McDonald AJ 1984).

3. The calculation for the LFP needs to be better justified in relation to previous studies.

To address this, we empirically estimated the scaling factor for the LFP strength as a function of cell density. We systematically varied the cell density in the model and found how the LFP amplitude scaled as a function of it. This revealed that the LFP amplitude grows by the power of 0.67 as a function of cell density. All LFPs throughout the paper were replotted with the new scaling factor derived from this estimate. The methods section of the paper dealing with the LFP calculation has been rewritten to include the details of this calculation and make our choice of scaling factor clearer.

4. The novelty of the work should be better presented, and placed in context of similar work especially pertaining to gamma in other brain areas.

We added the following section to the discussion (starting line 922) to explain how our BL model of gamma is distinct from previous models of gamma based on cortical circuitry. We discuss how the microcircuitry of BL is not ideal for producing gamma oscillations, that our model is closer to a PING mechanism than the REI mechanism in visual cortex, and that unlike most other models we use a forward modeling approach to simulate how gamma appears in the LFP.

“BL microcircuitry presented unique challenges to gamma rhythmogenesis

The generation of gamma oscillations in our BL model was not a foregone conclusion. While qualitatively the BL network contains the microcircuitry required for producing gamma oscillations, quantitative differences in the properties of this network from previous PING models may have precluded gamma generation.

For a PING rhythm to emerge PNs must fire in advance of FSIs (Whittington et al. 2000), and thus increasing the average firing rates of PNs contributes to the strength of gamma oscillations produced in PING models and in slices (Adesnik and Scanziani 2010). PN firing rates in the BL are smaller than those in the cortical models used to simulate gamma by at least a factor of 6 (Börgers and Kopell 2008; Hoseini and Wessel 2016; Chariker et al. 2018), which may have diminished the network's ability to produce a PING type gamma rhythm. Also important is the strength of inhibitory feedback in the network (Börgers et al. 2012). Two factors that contribute to this are the strength of connections between PNs and FSIs and the number of FSIs in the network. We and others have found that the BL has a lower proportion of interneurons (McDonald and Mascagni 2001) compared with standard PING models (Börgers and Kopell 2008; Chariker et al. 2018, Hoseini and Wessel 2016). Moreover, the connection probability between PNs and FSIs in BL is lower (Woodruff and Sah 2007) than the aforementioned models. Thus, a priori the BL network may not be capable of producing gamma, and so our model was essential to at least test the possibility theoretically.

Another difference between our BL model and cortical-based PING models is a lower proportion of recurrent connectivity among PNs. Brunel and Wang (2003) found that including recurrent connections between excitatory units in a PING model could downshift the frequency of the network oscillation by half. On the other hand, our BL PING model has fairly little dependence on these connections, since their elimination did not substantially affect the prominence or peak frequency of gamma (Figure 4D). In addition, other models of gamma generation depend on the recurrent excitation among excitatory units. For instance, the REI model (Chariker et al. 2018) contains a network of reciprocally connected excitatory and inhibitory units. Unlike PING models, the gamma-periodic bursts of activity depend upon the buildup of recurrent excitation in the PN network, which raises the membrane potential for all units in the network, and drives them to fire synchronously. This mechanism for producing gamma is distinctly not PING, since the excitatory and inhibitory units activate simultaneously, instead of with the inhibitory units following the excitatory. Moreover, since BL PNs and FSIs in vivo fire during different phases of the gamma cycle (Amir et al. 2018), it is likely that the REI mechanism does not operate within the BL.

Thus, our model establishes the sufficiency of the BL network to produce gamma oscillations, and for those to arise via a PING mechanism. Despite that, it was also possible that any gamma produced by the model would be of insufficient power to comprise what is observed in the LFP in vivo. To address this, our model included a realistic estimation of the local field potential, which most PING models lack (Brunel and Hakim, 1999; Brunel and Wang, 2003; Borgers et al., 2005; Economo and White, 2012; Chalk et al., 2016; Hoseini and Wessel, 2016; Palmigiano et al., 2017; Traub et al., 2000; Bathellier et al., 2006; Neymotin et al., 2011; Chariker et al., 2018). Since our model LFP exhibited gamma oscillations that were close to the frequency and power of those recorded in vivo, it establishes the sufficiency of the local microcircuitry in BL to explain the gamma observed in LFP recordings in vivo.”

5. The relationship between the reported gamma to other frequencies needs to be better analyzed, both for bursts and entrainment.

While we agree that other frequency bands could be explored with this model, we intentionally excluded analyzing them for several reasons. First, from the outset it was clear that in vivo the power spectrum only showed a consistent prominence in the mid-gamma frequency band, with all other frequencies following the 1/f decline in power. This suggested to us that there was a distinct physiological phenomenon happening in the mid-gamma band apart from the other frequencies, and this warranted targeted investigation. Second, a priori we had little reason to investigate frequencies above the gamma band (&gt;120 Hz) in BL since there was virtually no information indicating their importance to BL function, and neurons in the BL tended not to entrain to them (Amir et al. 2018). Third, once we ran the model we found that the strength of its LFP for frequencies below gamma, &lt;30 Hz, was substantially weaker than that seen in vivo. Explanations for why this is the case and what it tells us about the origin of those low frequency signals in vivo are discussed in the manuscript (see Emergence of gamma oscillations in the BL model in the Results section and also Emulation of BL gamma in the Discussion section). However, this divergence gave us little confidence in the meaningfulness of any results arising from them. Thus, we felt it was best to focus the paper on the mid-gamma band.

That being said, the Reviewer 2 raised several important points regarding our focus on gamma and whether the mid-gamma band in particular is distinct in its relationship with network events. To address this, we have incorporated an additional analysis into the manuscript (Figure 2F) and provided analyses for the reviewer that establish the uniqueness of mid-gamma (see our responses to Reviewer 2 questions 2 and 3).

6. The authors must provide their code as required by the

Journal policy: “Policy on Computer Code and Software

Computational models: New computational neuroscience computer code must be included with the initial submission as Extended Data to ensure reproducibility...”

We have uploaded the code to the submission site as 'Extended data'

The detailed comments of the reviewers are appended as they provide more details and bring up some useful additional points.

================================================================

-

Reviewer 1:

The paper reports a pioneering modeling study the a part of amygdala (basolateral complex). The model consists of 27000 neurons, modeled as 2-compartment or 3-compartment neurons, and interestingly and importantly the model predicts both spikes and local-field potentials (LFPs). I find the approach to be sound and the work very accomplished.

Thank you.

Comments to the Authors

The paper of Feng et al. reports a pioneering modeling study the a part of amygdala (basolateral complex). The model consists of 27000 neurons, modeled as 2-compartment or 3-compartment neurons, and interestingly and importantly the model predicts both spikes and local-field potentials (LFPs). I find the approach to be sound and the work very accomplished. Since I also find the questions addressed to be both important and timely, I am supportive of publishing of this work in eNeuro.

I have, however, some comments and questions that I would like the authors to address prior to publication:

1. I miss a figure of the anatomical organisation of the basolateral complex of the amygdala. This organization, in particular on the geometric organisation of the dendrites of the different neurons, could have a strong effect on the LFP.

A new series of panels has been included (Figure 1A,B) illustrating the distribution of FSIs and PNs throughout the model, along with the random orientation of their processes and spatial extent (Figure 1C)

2a) On l. 301 it is stated that the dendrites of the neurons are randomly oriented. Is this what is observed in the amygdala? (I believe that in other nuclei the dendrites could have a radial organisation.)

Neurons in BLA do not have a layered structure as in the cortex, and the dendrites of PNs orient randomly (McDonald 1984). For this reason, we assumed random orientation for the dendrites. The relevant sentence has now been updated to include the reference (line 301):

“The dendrites of all neurons in the network had random orientations (McDonald, 1984).”

2b) If not, I would suggest that other geometrical configurations of the dendrites also should be considered. This may not only affect the overall amplitude of the LFP, but also the shape of LFP power spectra. This follows from the observation that (i) correlated synaptic input combined with (ii) spatially aligned dendrites can give a boost of the LFP at low frequencies (see Leski et al, PLoS Comp Biol 2013)

See answer to 2a) above.

2c) This could maybe contribute to an explanation for why the low-frequency powe of the LFP is smaller in the model than what is seen in experiments? (as mentioned on l. 564 and in the Discussion)

Please see responses to parts 2a) and to 2d) below.

2d) Note that the lack of low-frequency power in predicted LFP was also observed in models for cortical LFP, see Hagen et al, Cerebral Cortex (2016). It would be good if a connection to this previous study could be made.

This is again a good suggestion. We have modified the text, including both references, starting line 605, as follows:

“However, the model's spectrum had substantially less power below 30 Hz. This may reflect the absence of volume conducted activity or a lack of interactions with extrinsic structures. The former possibility was studied by Leski et al., 2013, who showed that lower-frequency LFP components have a larger spatial reach and extended further outside the active population than high-frequency components. It has also been found that increasing the correlations between extrinsic afferents boosts power in the low frequency band (Hagen et al. 2016; Leski et al. 2013). Our model likely underestimates the contribution of correlated extrinsic inputs since our naturalistic spike trains only represented a fraction of the structures projecting to the BL, each presumably with their own correlated spiking. Consistent with this, we found that replacing the naturalistic correlated spiking processes of our extrinsic afferents with rate matched independent Poisson processes further reduced the power of low frequencies (Figure 2B, green line). Importantly, gamma power from 50 to 80 Hz was unaffected by this change, supporting the hypothesis that it is locally generated and not imposed by upstream structures or volume conducted from adjacent regions.”

Leski S, Linden H, Tetzlaff T, Pettersen KH, Einevoll GT (2013) Frequency dependence of signal power and spatial reach of the local field potential. PLoS Comput Biol 9:e1003137.

Hagen E, Dahmen D, Stavrinou ML, Linden H, Tetzlaff T, van Albada SJ, Grun S, Diesmann M, Einevoll GT (2016) Hybrid Scheme for Modeling Local Field Potentials from Point-Neuron Networks. Cerebral cortex 26:4461-4496.

3. On l. 424 it is assumed that the LFP is proportional to the density of neurons. Is there a justification for this? In, for example Linden et al, Neuron (2011), it was found that in some network regimes (low synaptic input correlation) the LFP scales like the square root of the density while with large synaptic input correlation the scaling was more linear.

We agree with the reviewer and have addressed this issue in the revision. Specifically, we have revised the text, as follows (starting line 444):

“To correct for this, we rescaled the LFP by a correction factor that was estimated following Linden et al. (2011) who suggest that LFP scales as the square root of the number of neurons, N, for uncorrelated synaptic inputs. However, that scaling factor was derived from a network of pyramidal neurons positioned in a disk with uniformly oriented dendrites, and was not for density per se but actually the radius of the disk with density held constant. To determine the scaling factor in our model, we systematically varied the number of cells used to calculate the LFP from a full model run. Varying the cell count from 1000 up to 27000, we found that the standard deviation of the LFP scaled with density following N0.67. Since cell density in rats is reported to range from 2.5×104 to 2×105/mm3 (Tuunanen and Pitkanen, 2000; Salm et al., 2004; Pego et al., 2008; Rubinow and Juraska, 2009; Chareyron et al., 2011), while the model density is 9,840 neurons/mm3 (27,000 neurons, 1.4mm x 1.4mm x 1.4mm), the LFP correction factor would corresponding range from 1.9 to 7.5 [(〖2.5×〖10〗^4/9.84×〖10〗^3)〗^0.67 to (〖2×〖10〗^5/9.84×〖10〗^3)〗^0.67]. We chose the average of this range, 4.7, to scale the model LFP.”

Linden H, Tetzlaff T, Potjans TC, Pettersen KH, Grun S, Diesmann M, Einevoll GT (2011) Modeling the spatial reach of the LFP. Neuron 72:859-872.

Details for the reviewers on how to calculate the scaling factor: Linden et al. (2011) state that the scaling factor for predicting LFP amplitude is partly dependent on the correlation of synaptic inputs to cells in the network. We first calculated the correlation between the synaptic inputs using eqn. 15 in Linden et al. (2011) and found it to be 0.0012, which shows that it is close to being uncorrelated. We then used the model to estimate the dependence of LFP on the number of cells in our BL network (N). This was performed by first calculating the contribution to the LFP from all cells in the 27,000-cell network, and then iteratively dropping a random 1000 cells from the computation of the LFP, till the lower limit of N=1,000.

Such a process yielded the red circles (normalized to the value for the N=1,000 case) in the plot on the left. Blue and black dashed lines are for the linear, N or √N proportions, where N is number of cells in the network. These are again normalized to the N=1,000 case. This experiment allowed us to estimate the relationship between cell density in the model and changes in LFP amplitude.

A power law fit through the red curve shows the scaling factor to be 0.67, which is close to the scaling factor of 0.5 as reported in Linden et al. 2011. This is consistent with the low average correlation coefficient from our network that we calculated above.

Minor comments:

l. 71: anatomically detailed -&gt; anatomical realistic

Done.

l. 148: referral to “one dataset whose manuscript is in preparation”. This seems unorthodox.

We have reworded it as “one unpublished dataset”.

l. 306-317: The used of “x” for product is unnecessary. I suggest to remove them throughout the manuscript.

We have removed “x” but used “*” at some locations where the potential for ambiguity might exist (e.g., eqns. 4-6).

l. 322: Must be stated that V is in units of mV (or whatever)

Done. We state that right after equation 1.

l. 332: The use of “*” for product s unnecessary. I suggest to remove them.

Please see response two items above.

l. 389: Give formula for Ornstein-Uhlenbeck process

We have added a new section with equations and table to explain the way we modeled the background inputs (starting line 387):

“Specifically, stochastic background input Ibck had two independent components, excitatory and inhibitory, for both PNs and FSIs, modeled as follows:

I_bck=g_e (t)(V-E_e )+g_i (t)(V-E_i )

where g_e (t) and g_i (t) are time-dependent excitatory and inhibitory conductances, respectively; E_e=0 mV and E_i=-75 mV are respective reverse potentials. The two are modeled as Ornstein-Uhlenbeck processes described below:

(dg_e (t))/dt=-1/τ_e [g_e (t)-g_e0 ]+√(D_e ) χ_1 (t)

(dg_i (t))/dt=-1/τ_i [g_i (t)-g_i0 ]+√(D_i ) χ_2 (t)

where g_e0 and g_i0 are average conductances, τ_e and τ_i are time constants, D_e and D_i are noise 'diffusion' coefficients, χ_1 (t) and χ_2 (t) are Gaussian white noise of zero mean and unit standard deviation (see Table 6 for these parameter values).

The above two stochastic differential equations can be numerically modeled by using the following update rule:

g_e (t+∆t)=g_e0+[g_e (t)-g_e0 ]exp⁡(-∆t/τ_e)+A_e N_1 (0,1)

g_i (t+∆t)=g_i0+[g_i (t)-g_i0 ]exp⁡(-∆t/τ_i)+A_i N_2 (0,1)

Where N_1 (0,1) and N_2 (0,1) are normal random numbers. A_e and A_i are amplitude coefficients with

A_e=√(∂_e^2 [1-exp⁡(-2∆t/τ_e)] )

A_i=√(∂_i^2 [1-exp⁡(-2∆t/τ_i)] )

Table 6 lists the parameter values used in the point-conductance model.”

Table 6. Parameters related to point-conductance model

l. 454: once -&gt; one

Done

l. 559: Can't anything be fit by a 1/f power law over a short frequency-range? What is meant?

A power law is scale invariant, meaning that it will have the same shape and coefficients irrespective of the range of values used for its calculation.

l. 590: How can the entrainment of rates have anything to do with extracellular potentials in this model?

An issue that frequently arises in the analysis of unit entrainment to oscillations in the LFP is the degree to which a neuron's own transmembrane currents contribute to the extracellular field and cause spurious coupling. The most common example of this is the contamination of extracellular spike waveforms into the gamma band, leading to an artifactually high entrainment between spiking and LFP gamma recorded on the same electrode. To contend with this, experimenters often record spiking on one electrode and the LFP on another. One could imagine a similar phenomenon occurring in our model, where the distance dependent decay in entrainment to gamma merely reflected a decrease in the contribution of a particular cell to the LFP. In our model because we have access to the exact contribution of each neuron to the simulated LFP, we measure how fast this decays and see if it matches or decays more slowly than the change in entrainment. This analysis revealed that a neuron's contribution to the LFP decayed more than twice as fast as its entrainment to gamma, indicating that the spurious kind of spike/field coupling mentioned above was unlikely.

=============================================================

-

Reviewer 2

The authors constructed a biophysically and anatomically detailed model of the basolateral amygdala (BL), which reproduced several properties of the network observed experimentally. In particular, this model generated transient gamma oscillations, as reported by these authors previously. Using this model, they characterized spatial spread of gamma as well as network and synaptic properties.

I found their model comprehensive, the questions well motivated, and analysis thorough. I have some questions and comments though, as described below.

We appreciate the comment.

1. Given that there are several detailed ING-PING type models of gamma oscillations, the authors should specify in more detail which of their results are new in terms of modeling. I understand that parameters have been optimized for BL, which is a new thing, but some results, such as bursty nature of gamma, have been shown in other brain areas. For example, the bursty nature of gamma has been described by Bob Shapley's group, who mentioned gamma as “filtered noise”, see Burns et al., 2010 and 2011, JNS, Xing et al, 2012, JNS. Their earlier models were more abstract (based on variants of Wilson-Cowan), such as Kang et al., 2010, JCN, but more recently they also have detailed biophysical models (see work of Chariker et al., 2018, JNS). As mentioned by the authors themselves, several other groups have also used ING-PING type models to describe gamma. At the very least, the authors need to have a detailed Discussion about their model versus others, and in particular, if there is anything new that has not been shown previously in other parts of the brain.

Please see our response to Synthesis statement #4 above where we list the additional text we have added to address this concern.

2. Intermittent bursts of gamma (Figures 2 and 6): the model appears to have intermittent bursts of power at all frequencies, as seen in Figure 2E. Is there anything particular about the gamma band? If the authors really want to emphasize the properties of gamma bursts, they should compare gamma bursts with bursts at other frequencies. In this regard, recently it has been suggested that the bursty nature of gamma actually arises due to the variability in the spectral estimator (Subhash Chandran et al., 2018, JNP), and that previous studies have underestimated the burst duration. If the authors really want to emphasize the burst properties (Figure 6), they should ensure that their results do not suffer from signal processing artifacts (perhaps by changing the filter setting and testing if that changes the parameters) and also compare the burst statistics with nearby frequency bands (say between 40-60 and 80-100 Hz).

We were not aware of the recent study by Subhash Chandran et al. (2018) when we submitted our manuscript. However, we checked the matching pursuit algorithm in Chandran et al and felt it may not be applicable to our study, since it detects gamma bursts in 1D waveforms while ours focus on 3D space in Figure 6. The large number of recording sites for our 3D LFP monitoring and the extended recording durations would also have made the matching pursuit algorithm computationally prohibitive. Moreover, since Subhash Chandran et al., 2018 didn't make any claims about whether the longer burst durations they detected had any functional significance, we opted to keep our results aligned with those from most of the prior studies (e.g. Xing et al. 2012, Amir et al., 2018).

As per the reviewer's suggestion, we did re-analyze burst duration in adjacent gamma bands to see if they differed from those found at mid-gamma. The results of that analysis can be found in the following plot.

Burst duration in the mid-gamma band, which was the focus of this study, is significantly longer than the other bands we processed, perhaps because bursts were more synchronized the in mid-gamma band. Note that the burst duration we calculated in our model is based on the watershed algorithm that identifies contiguous convex regions of elevated gamma power across three spatial and one temporal dimension (see Materials and Methods). The longer burst durations in the mid-gamma band stem from the higher synchronization of gamma bursts among each individual electrode detected.

However, the Chandran et al. paper did motivate us to tackle the issue of gamma burst durations earlier in the paper. Fortuitously, we used wavelets to perform spectral analyses of our simulated and in vivo LFPs in Figure 2. In the Chrandran et al. paper, it was reported that gamma burst durations derived from wavelets were on par with those uncovered by the matching pursuit algorithm for high-amplitude gamma bursts. Thus, we calculated the probability distributions of the mid-gamma bursts in our wavelet power spectrograms with respect to their durations and amplitudes (Figure 2F). This revealed that the distribution for our model gamma bursts matched those recorded in vivo. Additionally, the mean duration for higher amplitude bursts (greater than 80th percentile in amplitude) was ~30 ms shorter than those from Figure 6. This could have arisen because gamma bursts can move throughout the BL, and so a single recording site is less likely to be both the origin and end point of a gamma burst, and thus will only 'see' the burst for a portion of its total existence.

Chandran Ks S, Seelamantula CS, Ray S (2018) Duration analysis using matching pursuit algorithm reveals longer bouts of gamma rhythm. Journal of neurophysiology 119:808-821.

Xing D, Shen Y, Burns S, Yeh CI, Shapley R, Li W (2012) Stochastic generation of gamma-band activity in primary visual cortex of awake and anesthetized monkeys. The Journal of neuroscience : the official journal of the Society for Neuroscience 32:13873-13880a.

3. Gamma entrainment (Figure 3): I was surprised that the authors only focused on the gamma band instead of comparing the phase locking etc in the entire frequency range. For example, is the phase locking shown in Figures 3B and 3C unique to gamma band? What about the real data? The authors should show spike-field coherence or PPC for both real and model data here before dissecting the network and cellular mechanisms in later figures (in which some of these analyses are done).

Please see our response to the Synthesis statement #5. Briefly, we focused on mid-gamma given its prominence in vivo and deficiencies in the interpretations of frequencies above and below the gamma band. While we strongly feel that including analyses outside the mid-gamma band would overwhelm an already packed paper and detract from its focus, we are more than willing to share with the reviewer the requested analysis.

These are the circular r values for the data from the Amir et al. 2018 study, which recorded from the BL in vivo, and several runs of our model with different random seeds. As you can see, for the most part the entrainment values predicted by the model in each frequency band fall within the distribution of values observed in vivo. Even though the results are concordant, we are still hesitant to include them because (1) we have reservations about the physiological interpretation of the low-frequency activities observed in our model and (2) frequencies above the gamma band (&gt;100 Hz) have yet to be ascribed to particular functions in the BL.

4. Figure 5C shows an increase in coherence for the PN population at high frequencies. Any idea why that is the case?

The increase in coherence above 100 Hz reflects the fact that IPSCs tended to follow EPSCs. In PNs because the EPSCs are so sparse, there is a strong lagged coordination between the onset of the EPSC and IPSC. Such coordination is much weaker in for FSIs. This can be seen in the figure below:

This is something we should have again recognized and addressed, and do so now. Since this effect is largely trivial, and the signal power was low at frequencies above 100 Hz, and so have limited the relevant plots in Fig. 5 to 100 Hz.

5. Figure 7G - when the network does not produce gamma, the peaks/troughs etc have no meaning, right? So it is surprising that no difference in spike probability is observed?

Even when the LFP lacks gamma oscillations it can still have peaks and troughs in the gamma band, since once can always filter the LFP in the frequency range of mid-gamma. Since the LFP reflects transmembrane currents, and in general neuronal depolarization will produce an LFP trough, while hyperpolarization will produce a peak, it could have been that the coordination we observed in figure 7G was merely reflecting this phenomenon irrespective of gamma. Consequently, Figure 7G was used to show that when gamma oscillations were present in BL, their troughs were the most sensitive part for generating spikes. By contrast, when BL lacked gamma oscillations, this significantly diminished the LFP phase dependent sensitivity of spikes to EPSCs (also compare Y axis on two panels), even though firing rates were unchanged. We believe that this analysis demonstrates that gamma oscillation are critical in the BL for the pacing the sensitivity of PNs to extrinsic inputs, and consequently synchronizing their spiking so that downstream targets are more effectively driven.

Minor

1. Please label A1 and A2 in Figure 1.

Done. Although now that the model geometry has been included in Figure 1, the corresponding panels are now D1 and D2.

2. Line 1307: “with” the effect, not “which”.

Done.
