# Supplementary material for: Gamma Oscillations in the Basolateral Amygdala: Biophysical Mechanisms and Computational Consequences
Source: eNeuro. 2019 Feb 5;6(1):ENEURO.0388-18.2018. doi: 10.1523/ENEURO.0388-18.2018 (PMC6361623; doi:10.1523/ENEURO.0388-18.2018)
Supplement: Supplementary Material 1 — Supplementary Code used in article. Download Supplementary Material 1, ZIP file [file sup_enu-eN-NWR-0388-18-s01.zip › sent to eNeuro/readme.rtf]

This is the readme for the model associated with the manuscript:
Gamma oscillations in the basolateral amygdala: biophysical mechanisms and computational consequences
This model was contributed by Feng Feng.
This is the first large-scale biologically realistic model of the basolateral amygdala (BL) with parameters constrained by published literature. The model matches closely with numerous in vivo testing results, including average neuron firing rate, gamma oscillation detected on LFPs, and firing phase entrainments to gamma oscillation with spatial gradients. 
This model has been developed using the NEURON simulator (Hines and Carnavale, 2008).
Summary of files: 
DATA INPUT FILES:
spikesmatrix_op:   Stores spikes for each external connection (row).
Cell_type.txt:      This file indicates the type of cell for the PNs and FSIs. The types are defined in the main file. 1 for A PNs, 10 for C PNs and 100 for FSIs.
active_syn_op:     Stores precell ids for each postcell (row), for internal connections.
active_syn_GAP_op: Stores connected FSI IDs via gap couplings.
sim_length:        Simulation time length.
location.txt:        Stores 3D coordinates (um) for each cell
oritation.txt:        Stores dendrite orientations for each cell
elec_coords.txt:     List electrode coordinates (um) to calculate LFPs 

 NEURON FILES:
- BL_main.hoc:       Main file to run the simulations
- function_ConnectInputs_invivo_op.hoc:  Establish external connections and feed predefined external spikes to BL network with adjustable synapse weight parameters.
- function_ConnectInternal_simplify_online_op.hoc: Establish internal connections with adjustable synapse weight parameters.
- function_ConnectInternal_gj_simplify: Establish gap coupling between FSIs with adjustable coupling strength parameters.
- function_ConnectInternal_simplify_online_op.hoc: Establish internal connections with adjustable synapse weight parameters.
- function_ConnectTwoCells.hoc: Procedure used to connect two internal neurons.
- function_calcconduc: Function used to calculate conductances for being used to calculate LFPs. 

OUTPUT FILES:
data saves spiking time history of all cells
\LFPs\LFP_elec_0: saves calculated center electrode LFP.

BEFORE YOU RUN:
Make sure to make an empty folder named LFPs in the directory. NEURON will save the calculated LFP files into this directory.
